# Functional trait divergence and trait plasticity confer polyploid advantage in heterogeneous environments

**DOI:** 10.1111/nph.15508

**Published:** 2018-10-27

**Authors:** Na Wei, Richard Cronn, Aaron Liston, Tia‐Lynn Ashman

**Affiliations:** ^1^ Department of Biological Sciences University of Pittsburgh Pittsburgh PA 15260 USA; ^2^ Pacific Northwest Research Station United States Department of Agriculture Forest Service Corvallis OR 97331 USA; ^3^ Department of Botany and Plant Pathology Oregon State University Corvallis OR 97331 USA

**Keywords:** adaptation, adaptive plasticity, common gardens, functional traits, polyploidy, wild strawberry

## Abstract

- Polyploidy, or whole‐genome duplication often with hybridization, is common in eukaryotes and is thought to drive ecological and evolutionary success, especially in plants. The mechanisms of polyploid success in ecologically relevant contexts, however, remain largely unknown.
- We conducted an extensive test of functional trait divergence and plasticity in conferring polyploid fitness advantage in heterogeneous environments, by growing clonal replicates of a worldwide genotype collection of six allopolyploid and five diploid wild strawberry (*Fragaria*) taxa in three climatically different common gardens.
- Among leaf functional traits, we detected divergence in trait means but not plasticities between polyploids and diploids, suggesting that increased genomic redundancy in polyploids does not necessarily translate into greater trait plasticity in response to environmental change. Across the heterogeneous garden environments, however, polyploids exhibited fitness advantage, which was conferred by both trait means and adaptive trait plasticities, supporting a ‘jack‐and‐master’ hypothesis for polyploids.
- Our findings elucidate essential ecological mechanisms underlying polyploid adaptation to heterogeneous environments, and provide an important insight into the prevalence and persistence of polyploid plants.

## Introduction

Polyploidy (or whole‐genome duplication often with hybridization) results in heritable occurrence of more than two sets of chromosomes of the same (autopolyploidy) or disparate origins (allopolyploidy), which enlarges and diversifies an organism's genome with profound influence on phenotype and fitness (Otto & Whitton, 2000; Ramsey & Ramsey, 2014; Soltis *et al*., 2016). While polyploidy is common in eukaryotic lineages, some of the best‐known examples of polyploids in flowering plants include important crops (Salman‐Minkov *et al*., 2016) and many invasive species (te Beest *et al*., 2012), and the repeated and pervasive occurrence of polyploidy throughout the plant kingdom reflects its widespread adaptive significance (Van de Peer *et al*., 2017). Despite its evolutionary importance, the mechanisms of polyploid advantage in ecological contexts are largely unknown. A leading, yet rarely tested, hypothesis is that polyploid fitness advantage arises from altered phenotype (i.e. functional trait divergence from diploids) and/or enhanced ability to adjust phenotype (i.e. functional trait plasticity) in response to environmental change (Levin, 1983; Van de Peer *et al*., 2017).

Polyploidy can alter plant phenotype (Levin, 1983; Soltis *et al*., 2014). Phenotypic variation at the cellular level (e.g. an increase in cell size) as a result of an increase in ploidy was first recognized in early cytological studies of synthetic polyploids (reviewed in Ramsey & Ramsey, 2014). This positive correlation between genome size and cell size holds across angiosperm lineages (Masterson, 1994; Beaulieu *et al*., 2008), whereas for phenotype at higher levels (e.g. tissues or organs), the nucleotypic effects of genome size are shown to be weaker or absent (Knight & Beaulieu, 2008). In addition to the genome size effect, polyploidy can also diversify a plant genome by incorporating multiple copies of genes from the same or different species, which can have important implications for phenotype (Chen, 2010; Soltis *et al*., 2014). Comparisons of functional trait divergence between diploids and naturally occurring polyploids, in ecologically relevant contexts, have been primarily conducted in autopolyploids with intraspecific ploidal variation (Ramsey & Ramsey, 2014), and have yielded mixed, and often species‐specific, conclusions (e.g. Li *et al*., 1996, 2012; Maherali *et al*., 2009; Balao *et al*., 2011; Hao *et al*., 2013). The phenotypic consequences on functional traits of allopolyploidy – which generates diverse genetic backgrounds and the potential to express transgressive phenotypes relative to autopolyploidy (Chen, 2010) – remains unclear for the vast majority of wild allopolyploid taxa that account for half of the extant polyploids (Barker *et al*., 2016), with few exceptions (Buggs & Pannell, 2007; Hahn *et al*., 2012; Manzaneda *et al*., 2015; Leal‐Bertioli *et al*., 2017).

Polyploidy has the potential to alter functional trait plasticity (referred to as trait plasticity hereafter), owing to genomic redundancy and versatility in gene expression (Stebbins, 1971; Adams & Wendel, 2005; Leitch & Leitch, 2008; Jackson & Chen, 2010; Madlung & Wendel, 2013). Relative to diploids, polyploids can potentially employ alternative copies of duplicated genes gained from diverse and possibly adaptive genetic backgrounds to respond to novel environments (Bardil *et al*., 2011; Dong & Adams, 2011; Shimizu‐Inatsugi *et al*., 2017). Thus, it is hypothesized that polyploids can exhibit higher trait plasticity than diploids in response to varying environment. Previous work has primarily emphasized gene expression changes of polyploidy (Soltis *et al*., 2016), and, as a result, the questions of whether genome duplication translates into increased trait plasticity in the wild (Madlung, 2013), and how trait plasticity differs between diploids and polyploids (Buggs & Pannell, 2007; Hahn *et al*., 2012; Manzaneda *et al*., 2015), persist.

Polyploidy has been demonstrated to provide selective advantages to plants under environmental stresses and instabilities (Chao *et al*., 2013; Yang *et al*., 2014; Van de Peer *et al*., 2017). However, it remains controversial whether such polyploid fitness advantage occurs only in a particular environment or can be maintained consistently across environments (Ramsey, 2011; Madlung, 2013). Several competing adaptive hypotheses, based on the fitness reaction norm extended from theories of invasion (Richards *et al*., 2006), have been proposed. First, elevated genetic heterozygosity and gene expression versatility may enable polyploids to occupy broader ecological niches (i.e. higher ecological amplitude) than diploids. As a result of possessing such ‘general purpose’ genotypes (Baker, 1965; Stebbins, 1971), polyploids could exhibit high fitness and fitness homoeostasis (i.e. constant fitness) in heterogeneous environments (manifesting as high intercept and low slope in a fitness reaction norm; ‘jack‐of‐all‐trades’) (Richards *et al*., 2006). Alternatively, in the absence of fitness homoeostasis, polyploids may still maintain higher fitness than diploids across a broad range of environments. This fitness strategy (manifesting as high intercept and high slope) can be referred to as ‘jack‐and‐master’ (Richards *et al*., 2006). Lastly, polyploids and diploids may both be habitat specialists, exhibiting high fitness in alternative environments (i.e. ‘master‐of‐some’). While these adaptive hypotheses have been tested among invasive and native plant species (e.g. Richards *et al*., 2006; Davidson *et al*., 2011), tests with respect to polyploidy are not only limited to a few intra‐ and interspecific systems between diploids (2*n* = 2*x*) and mostly tetraploids (2*n* = 4*x*) (Petit & Thompson, 1997; Bretagnolle & Thompson, 2001; McIntyre & Strauss, 2017), but more importantly these lack the mechanisms that connect the fitness of diploids and polyploids to functional traits and trait plasticity.

**Figure 1:**
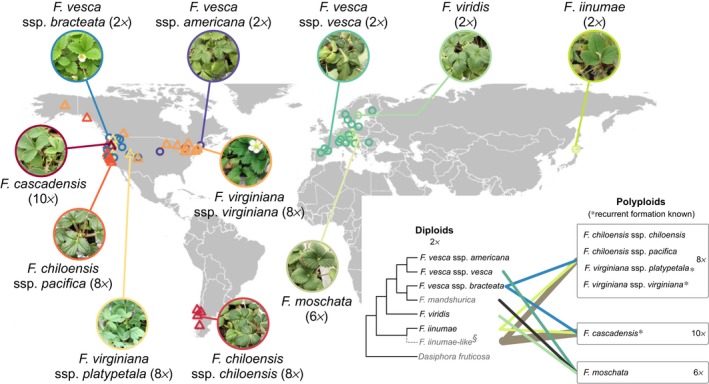
Seventy‐two source populations of diploid (circles) and polyploid (triangles) *Fragaria*, and their reticulate evolutionary histories (inset). The inset dendrogram represents the known evolutionary relationships among the five diploid (2*x*) taxa in this study (black), as well as those not in this study (grey; *F. mandshurica*, and an extinct *F. iinumae*‐like diploid^§^ with dashed branch), along with an outgroup taxon (*Dasiphora*). Among the six polyploids, the octoploid (8*x*) taxa are derived from the 2*x F. vesca* ssp. *bracteata*,* F. iinumae* and the extinct *F. iinumae*‐like diploid (each contributing, respectively, two, two and four sets of chromosomes to the 8*x* genomes, reflected by line width) (Tennessen *et al*., 2014; Wei *et al*., 2017a). The 10*x F. cascadensis* has two sets of chromosomes from *F. vesca* ssp. *bracteata*, two sets from *F. iinumae* and six sets from the *F. iinumae*‐like diploid (Wei *et al*., 2017b). The 6*x F. moschata* is derived from *F. vesca* ssp. *vesca*,* F. viridis* and *F. mandshurica* (Kamneva *et al*., 2017). Recurrent formation* of the same polyploid taxon in different populations has been previously identified (Dillenberger *et al*., 2018), whereas such information remains unclear for the remaining polyploid taxa.

In this study, we take advantage of the fact that polyploidy is an important mode of speciation in wild strawberries (*Fragaria* L.), a genus that originated around 3–8 million yr ago (Liston *et al*., 2014; Qiao *et al*., 2016) and has 22 extant species with a broad distribution in the northern hemisphere (Staudt, 1999; Liston *et al*., 2014). While *Fragaria* has two centers of species diversification (in East Asia and Europe–North America; Liston *et al*., 2014), we focused on diploid and polyploid *Fragaria* that occur in North America, South America, Europe and Northeast Asia (Fig. 1), among which repeated and independent events of allopolyploid speciation (Fig. 1) have been revealed by polyploid *Fragaria* genomes (Tennessen *et al*., 2014; Kamneva *et al*., 2017; Wei *et al*., 2017a,b; Dillenberger *et al*., 2018).

By growing clonal replicates of a worldwide collection of *Fragaria* genotypes of five diploid and six allopolyploid taxa (2*n* = 6*x*–10*x*, primarily 8*x*; Fig. 1; Supporting Information Table S1) in three climatically different common gardens in Oregon, USA, we addressed the following questions: do functional traits differ between diploids and polyploids; do polyploids demonstrate higher trait plasticity than diploids in response to environmental change; is there a polyploid fitness advantage across diverse garden environments; and, if so, is the polyploid fitness advantage conferred by trait means or trait plasticities, or both?

## Materials and Methods

### Study system

*Fragaria* are perennial herbaceous plants that reproduce both sexually by seed and asexually by plantlets on stolons (Staudt, 1999). The six allopolyploid strawberries studied here are hexaploid (6*x*) *F. moschata*, octoploid (8*x*) *F. chiloensis* ssp. *pacifica*,* F. chiloensis* ssp. *chiloensis*,* F. virginiana* ssp. *platypetala*,* F. virginiana* ssp. *virginiana*, and decaploid (10*x*) *F. cascadensis*. The five diploid strawberries are *F. vesca* ssp. *bracteata*,* F. vesca* ssp. *americana*,* F. vesca* ssp. *vesca*,* F. viridis*, and *F. iinumae*. We defined ploidy level broadly as diploid or polyploid, owing to distinct separation between diploids and high‐order polyploids (2*n* ≥ 6*x*; Fig. S1), and the dominance of the 8*x* taxa and genotypes within high‐order polyploids (Table S1). Among these polyploids, the 10*x* and 8*x* taxa are derived from the 2*x F. vesca* ssp. *bracteata*,* F. iinumae* and an extinct *F. iinumae*‐like 2*x* taxon (Fig. 1; Tennessen *et al*., 2014; Wei *et al*., 2017a,b), and the 6*x* is derived from the 2*x F. viridis* and *F. vesca* ssp. *vesca* as well as the Asian 2*x F. mandshurica* (Fig. 1; Kamneva *et al*., 2017). Recurrent formation, representing multiple independent origins, has been observed in the 10*x* and 8*x* taxa (Dillenberger *et al*., 2018). The worldwide collection of *Fragaria* was conducted as an international collaborative effort in 2013–2014; details are available on our Wild Strawberry website (http://wildstrawberry.org/; see also Fig. S2).

### Genotype and clone cultivation

In April 2015, we germinated and grew four genotypes (i.e. each from a single, open‐pollinated seed of a distinct wild plant) from each of 72 total populations across the 11 taxa (10 populations of less than four genotypes; Table S1), in a glasshouse at the University of Pittsburgh following standard protocols (Wei *et al*., 2017b). In September 2015, we harvested 12 plantlets (clones) from stolons of each of the 269 genotypes (24 genotypes of < 12 clones; Table S1). These clones (*N *=* *3137) were sent to Oregon State University, kept in the dark at 16°C for 1 wk to stimulate root growth, and then transplanted to 107 cm^2^ conetainers (Stuewe & Sons Inc., Tangent, OR, USA) filled with Sunshine Mix #4 soil (Sun Gro Horticulture, Agawam, MA, USA). Plantlets were grown at 18°C under natural lighting in a glasshouse for 3 wk, and moved outside for 1 wk before transplanting in common gardens during autumn (28 October to 15 November 2015). At transplanting, clones had one to two leaves, most of which senesced over winter.

### Common gardens

**Figure 2:**
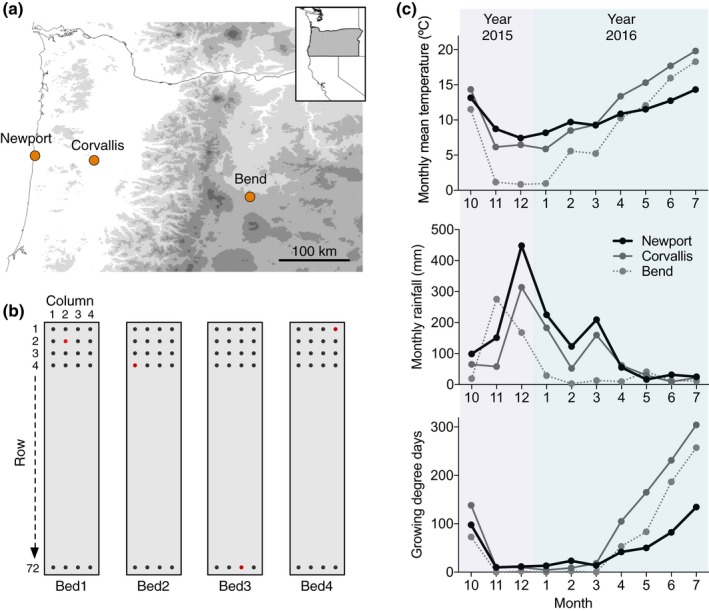
The location, design and climate of common gardens. (a) Three common gardens were located in Oregon, USA, including the coastal garden at Newport, the valley garden at Corvallis and the montane garden at Bend. (b) We established four raised wooden beds at each garden location. Each bed (18 × 1.5 m) can host 72 × 4 plants, indicated by the dots. For each genotype, the four clones (red dots) were assigned to the four beds, and the position within each bed was chosen randomly. (c) The monthly mean temperature, rainfall and growing degree days (i.e. the cumulative heat > 10°C) were obtained (see Supporting Information Methods S1) for the three common gardens, during the course of the field experiment from October 2015 to mid‐July 2016.

Three common gardens were located in Oregon, USA (Fig. 2a): cool/coastal ‘Newport’ (44.62046°N, 124.04410°W; altitude, 5 m), temperate/valley ‘Corvallis’ (44.56107°N, 123.28911°W; 70 m) and arid/montane ‘Bend’ (44.08895°N, 121.26192°W; 1063 m), each differing in temperature, precipitation and soil (Fig. 2c; Table S2). At each location, we established four raised wooden beds (18 × 1.5 m; Fig. 2b), filled with soil derived from local sources (Methods S1; Table S2).

Plants were arranged in a complete randomized block design with *c*. 25 cm spacing, and one clone per genotype was randomly assigned a position in one of the four beds (blocks) at each garden location (Fig. 2b). For the 24 genotypes with < 12 clones, we distributed available clones evenly across garden locations, but within each garden we prioritized filling beds 1 and 2 to have at least two complete blocks each location. Empty positions (*N *=* *319) were filled with nonexperimental clones, which were cultivated in the same manner as the others, to maintain even plant spacing and density. Throughout the course of the experiment (October 2015–July 2016), plants received only natural precipitation at Newport and Corvallis, which reached a total of 138.5 and 95.5 cm, respectively (Fig. 2c); however, at Bend (natural precipitation of 58.2 cm), plants were given supplemental water totaling 14.2 cm during the months of near‐zero rainfall (February–April 2016; Fig. 2c). All beds were protected from large herbivores using polypropylene mesh (1.6 × 1.6 cm) netting. Beds at Bend received straw cover (November 2015–February 2016) to minimize winter freeze damage to plant crowns.

Given the amelioration of freezing and drought stress at Bend, we considered Corvallis the most favorable environment, and Newport the least favorable based on growing degree days (i.e. the cumulative heat > 10°C; Fig. 2c).

### Functional traits and fitness components

We assessed a suite of leaf functional traits that capture essential plant ecophysiological processes (Table 1) in May 2016 on experimental plants in beds 1 and 2 of each garden (*N *=* *1429). We counted the number of leaves of individual plants, and collected the largest, fully expanded leaf over a 14 d period for all selected beds to measure leaf area and seven functional traits as described in Methods S1. Among these traits, vein density and trichome density were measured only at Corvallis and Bend (*N *=* *950), as the collected leaves from plants at Newport were too small for these additional measurements (see Methods S1). Leaf nitrogen content and carbon isotope discrimination were obtained for a subset of randomly chosen genotypes per population at individual gardens (*N *=* *210).

**Table 1:** Key variables of the common garden experiment

	Description (unit)	Function^a^
Leaf functional traits		
Specific leaf area (SLA)	Light‐capturing leaf area per unit dry mass (mm^2^ mg^−1^)	SLA reflects the thickness and/or dry mass content of leaf tissue. High SLA permits high leaf carbon gain.
Stomatal density (SD)	Abaxial stomata per unit leaf area (mm^−2^)	SD regulates CO_2_ intake and water transpiration, reflecting the tradeoff between gas conductance and epidermal construction cost.
Stomatal length (SL)	Abaxial guard cell length (μm)	SL regulates CO_2_ intake and water transpiration. SL correlates negatively with SD.
Vein density (VLA)	Total minor vein lengths per unit leaf area (mm mm^−2^)	VLA supports leaf hydraulic conductance. Low VLA, however, reduces construction cost.
Trichome density (TD)	Total abaxial and adaxial trichomes per unit leaf area (mm^−2^)	TD influences the ability of plants to prevent water loss.
Nitrogen content (*N*_mass_)	Leaf nitrogen per unit dry mass (%)	*N*_mass_, required for photosynthetic proteins, supports leaf photosynthetic potential.
Carbon isotope discrimination (Δ^13^C)	Amount of isotope discrimination against ^13^C relative to ^12^C during photosynthesis (‰)	Δ^13^C reflects photosynthetic water‐use efficiency, integrated over the life span of a leaf. Low Δ^13^C indicates high water‐use efficiency.
Plant fitness components		
Survival	Presence (1) or absence (0) of a plant	It is used to estimate genotypic survival rate here.
Plant size	Leaf number × leaf area (dm^2^)	It reflects plant growth since transplanting.
Stolon mass	Dry mass of stolons (g)	It reflects asexual reproduction.

a References for trait function: SLA (Poorter *et al*., 2009); SD and SL (Hetherington & Woodward, 2003); VLA (Sack & Scoffoni, 2013); TD (Ehleringer & Björkman, 1978; Sletvold & Ågren, 2012); *N*_mass_ (Wright *et al*., 2004); Δ^13^C (Farquhar & Richards, 1984).

We scored plant survival in May 2016 on plants in all four beds each garden. For plants in beds 1 and 2 of each garden, we estimated plant size as the product of leaf number and the area of the largest leaf (Table 1). For reproduction, as most plants did not flower in 2016, we focused on asexual reproduction (stolon mass). All experimental plants survived to the time (7–14 July 2016) when we harvested stolons, which were dried at 65°C for 1 wk before weighing.

### Climatic niche distance

Plant functional traits and fitness can be influenced by climatic differences between source populations and experimental gardens (Rehfeldt *et al*., 1999), or the ‘climatic niche distance’ (CND). To estimate CND, we extracted the 19 bioclimatic variables (current conditions, 1970–2000) at 30 arcsec resolution (or 2.5 arcmin resolution for west coast populations of North America), as well as altitude estimates, from worldclim v2.0 (Fick & Hijmans, 2017) for the 72 source populations and the three garden locations. We conducted a principal component analysis (PCA) of these 20 variables using prcomp in R v3.3.3 (R Core Team, 2017). The first five PCs, accounting for 94.2% of the variation (Fig. S3), were used to calculate the Euclidean CND between each source population and each garden using the R package pdist (Wong, 2013). Owing to the lack of soil data from source *Fragaria* populations, our estimates of CND did not include soil variables.

### General linear mixed models

We addressed each of the four questions in the Introduction using linear mixed models (LMMs) with the package lme4 (Bates *et al*., 2015). While the response variables and predictors (fixed effects) of LMMs were specific to each question, all LMMs accounted for evolutionary dependence among populations and taxa using nested random effects (i.e. populations nested in taxa and taxa in ploidy level, ploidy/taxon/population), which outperformed phylogenetic LMMs based on *Fragaria* plastid tree (Fig. S4) that simplifies the reticulate relationships (Fig. 1) between diploid and polyploid taxa (see Methods S1; Figs S5, S6). Moreover, for all LMMs, response variables were power‐transformed using the Box–Cox method in the package car (Fox & Weisberg, 2011) to improve normality, and the absence of multicollinearity among predictors was confirmed using the variance inflation factor. We evaluated the statistical significance (by type III sums of squares) of predictors and their least‐squares means in LMMs using the package lmertest (Kuznetsova *et al*., 2017). The variance explained by predictors and random effects of LMMs was assessed using the package mumin (Bartoń, 2017).

To evaluate whether diploids and polyploids differ in functional traits (the first question), the response variables of LMMs considered genotypic values of each functional trait (i.e. the average of two clones) at each garden. The predictors included central leaflet width + CND + garden + ploidy + ploidy : garden + ploidy : CND. We incorporated central leaflet widths, which were similar among diploid and polyploid taxa (Fig. S7), to account for functional trait variation potentially attributable to leaf characteristics (e.g. expansion, vigor), despite using a standardized leaf collection protocol as described in the section ‘Functional traits and fitness components’.

To evaluate whether polyploids express higher trait plasticity than diploids (the second question), we estimated plasticity for each trait and genotype using relative distance plasticity index (RDPI) and phenotypic plasticity index (PI; Valladares *et al*., 2006). For traits that were only measured at two gardens (vein density and trichome density), plasticity was calculated as trait distance (in absolute value) of the same genotype between the two environments, divided by the mean (for RDPI) or by the maximum (for PI) of the two genotypic trait values. For traits measured at all three gardens, RDPI and PI were calculated as the mean of the three pairwise distances. The response variables of LMMs considered genotypic plasticity of each trait, and the predictors included CND mean (i.e. genotypic CND averaged across gardens) + ploidy + ploidy : CND mean.

To evaluate whether polyploids have higher fitness than diploids (the third question), we estimated genotypic fitness of these perennial plants at each garden using a composite fitness index as the multiplicative product of genotypic survival rate, growth (plant size) and asexual reproduction (stolon mass). The genotypic survival rate was calculated as the proportion of clones that survived to May 2016 in all four beds per garden. The genotypic plant size and stolon mass were the average of the two clones measured per garden. As many plants produced zero stolons at Newport, we adjusted the genotypic stolon mass at each garden by adding 0.01 g. Our estimate of fitness represented a relatively equal contribution from each of the three components, given their similar scales across gardens (Fig. S8). Fitness (with the power transformation parameter *λ* = 0.1) was taken as the response variable, and the predictors of the LMM included CND + garden + ploidy + ploidy : garden + ploidy : CND.

To determine whether plant fitness over all garden environments is associated with trait means or plasticities (the fourth question), we used genotypic average fitness (across gardens) as the response variable (power transformation, *λ* = 0.1) in LMMs. The predictors of each LMM included CND mean + trait plasticity + trait mean (i.e. genotypic trait averaged across gardens) + ploidy : trait plasticity + ploidy : trait mean, for each functional trait. Correlations between trait plasticity and trait mean were weak (Table S3). LMMs with trait plasticities of RDPI and PI were performed separately, but as they yielded similar patterns, we only reported the results based on RDPI. To compare the magnitude of the respective effects of trait mean and trait plasticity on average fitness and to assess how they differ between ploidy levels, we estimated the effect sizes (i.e. standardized coefficients, *β′*) of the predictors using the package sjplot (Lüdecke, 2017).

## Results

### Do functional traits differ between diploids and polyploids?

**Figure 3:**
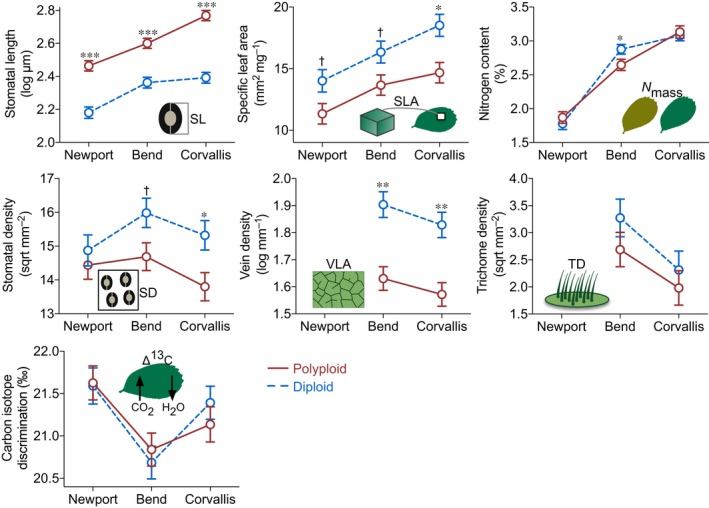
Diploid and polyploid *Fragaria* differ in leaf functional traits. The least‐squares mean ±1 SE of each trait are plotted for diploids (dashed lines) and polyploids (solid lines) at each garden location, estimated from general linear mixed models where the response variables were power‐transformed if necessary (see the Materials and Methods section; sqrt, square root; log, natural logarithm). The *x*‐axis is arranged from the least favorable, cool/coastal garden at Newport to the most favorable, temperate/valley garden at Corvallis. SL, stomatal length; SLA, specific leaf area; *N*_mass_, nitrogen content; SD, stomatal density; VLA, vein density; TD, trichome density; Δ^13^C, carbon isotope discrimination. VLA and TD were not available for plants at Newport. Significance levels: ***, *P *<* *0.001; **, *P *<* *0.01; *, *P *<* *0.05; †, *P *=* *0.053.

Diploid and polyploid *Fragaria* differed in most leaf functional traits (Fig. 3; Table S4), either consistently across environments (e.g. stomatal length and vein density) or in certain environments (e.g. specific leaf area, stomatal density and nitrogen content).

Polyploids possessed larger stomata (gauged by stomatal length; Table 1) than diploids in all environments (*F *=* *37.56, df = 1, *P *<* *0.001; Table S4). In the favorable environment at Corvallis, polyploids produced not only larger stomata (*post hoc* contrast of least‐squares means (LS means), *t *=* *8.48, *P *<* *0.001), but also fewer stomata per unit leaf area (*t *=* *−2.57, *P *=* *0.028) than diploids, which may lower the epidermal construction cost of stomata per unit area for gas exchange (de Boer *et al*., 2016) in polyploids. The general tradeoff between stomatal length and density seen across vascular plants (Franks & Beerling, 2009) was, nevertheless, decoupled in the stressful environment at Newport (Fig. 3); reduced stomatal length was not accompanied by increased stomatal density, for polyploids and especially diploids, which could negatively affect photosynthetic potential (Tanaka *et al*., 2013). Polyploids and diploids also differed in leaf vein density across environments (*F *=* *4.74, df = 1, *P *=* *0.037; Fig. 3; Table S4), with polyploids producing lower minor vein length per unit leaf area (i.e. lower hydraulic construction cost; Sack & Scoffoni, 2013).

Although the main effect of ploidy level across gardens did not influence specific leaf area (*F *=* *3.10, df = 1, *P *=* *0.100; Table S4) and nitrogen content (*F *=* *0.57, df = 1, *P *=* *0.453), polyploids produced foliage with significantly smaller specific leaf area compared with diploids at Corvallis (LS means contrast, *t *=* *−3.18, *P *=* *0.011), and significantly lower nitrogen content at Bend (*t *=* *−2.12, *P *=* *0.040). By contrast, polyploids and diploids were similar in leaf traits that influence water loss (trichome density, *F *=* *0.96, df = 1, *P *=* *0.346) and water‐use efficiency (carbon isotope discrimination, *F *=* *1.46, df = 1, *P *=* *0.233) in all environments (Fig. 3; Table S4).

### Do polyploids demonstrate higher trait plasticity than diploids in response to environmental change?

*Fragaria* genotypes expressed plasticity for the measured traits in response to different environments (Fig. 3), as demonstrated by the significant main effect of garden on each trait (all *P *<* *0.001; Table S4). Quantifying plasticity using RDPI and PI yielded similar patterns in degrees of plasticity among traits: carbon isotope discrimination had the lowest plasticity (mean RDPI = 0.02; PI = 0.05); stomatal length, stomatal density, specific leaf area and vein density exhibited fivefold higher plasticity (RDPI = 0.10, 0.13, 0.12 and 0.10, respectively; PI = 0.18, 0.26, 0.21 and 0.22, respectively); nitrogen content and trichome density had the highest (10‐fold) plasticity (RDPI = 0.21 and 0.36, and PI = 0.32 and 0.61, respectively). Yet, plasticity of these traits showed low correlation (Table S5), suggesting limited plasticity integration (Pigliucci, 2003) among traits. LMMs indicated that polyploids and diploids had similar degrees of plasticity for all seven traits (all *P *>* *0.05 for RDPI and PI; Table S6).

### Is there a polyploid fitness advantage across diverse garden environments?

**Figure 4:**
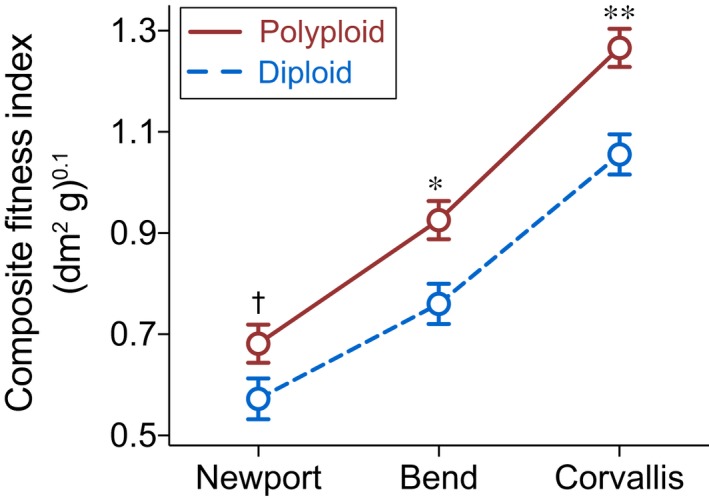
Polyploid *Fragaria* exhibit higher fitness compared with diploids. The composite fitness index, which was the product of genotypic survival rate, growth (plant size) and asexual reproduction (stolon mass), was transformed (with a power parameter *λ* = 0.1) in the general linear mixed model. The least‐squares means ±1 SE are plotted for diploids (dashed line) and polyploids (solid line) at each garden location. Significance levels: **, *P *<* *0.01; *, *P *<* *0.05; †, *P *=* *0.072.

The main effect of ploidy level influenced plant fitness (*F *=* *20.02, df = 1, *P *<* *0.001; Fig. 4; Table S7), after accounting for the significant negative effect of climatic niche distance (*F *=* *71.54, df = 1, *P *<* *0.001), as revealed by the LMM (*R* ^2^ of fixed effects = 0.61; Table S7). Polyploids had significantly higher fitness than diploids at Corvallis (LS means contrast, *t *=* *3.86, *P *=* *0.002) and Bend (*t *=* *3.02, *P *=* *0.011), and marginally higher at Newport (*t *=* *1.97, *P *=* *0.072), a pattern that refutes the ‘master‐of‐some’ strategy for polyploids or diploids. Fitness changed dramatically for both polyploids and diploids across the three gardens (garden effect, *F *=* *563, df = 2, *P *<* *0.001; Table S7), contradicting fitness homoeostasis of the ‘jack‐of‐all‐trades’ hypothesis but instead supporting the ‘jack‐and‐master’ hypothesis for polyploids.

### Is the polyploid fitness advantage associated with trait means or trait plasticities?

**Figure 5:**
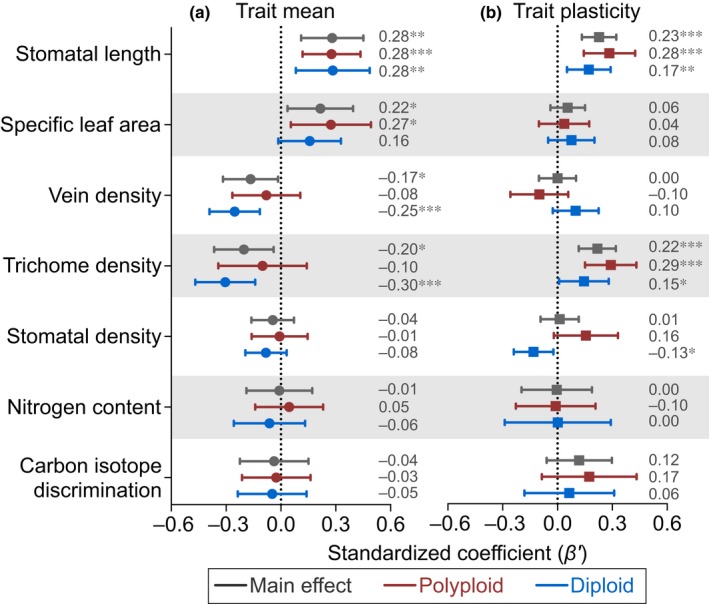
Trait means (a) and trait plasticities (b) predict average fitness of diploids and polyploids across the heterogeneous garden environments. Standardized regression coefficients (*β*′) of the main (grey) and ploidy‐specific (red, polyploid; blue, diploid) effects of trait means and plasticities on fitness are presented from general linear mixed models fitted separately for each functional trait. The average estimates of standardized coefficients are denoted by the symbols and the values to the right of the error bars (95% confidence intervals). Significance levels: ***, *P *<* *0.001; **, *P *<* *0.01; *, *P *<* *0.05.

For both diploids and polyploids, average fitness was influenced by mean values for four of the seven functional traits (i.e. stomatal length, specific leaf area, vein density and trichome density; grey colour, Fig. 5a; Table S8), with the magnitude of effect sizes often differing between ploidy levels (Fig. 5a). The trait mean of stomatal length had a significant positive effect on average fitness (Fig. 5a), indicating that plants having larger stomata were associated with higher fitness, and the magnitude of this positive effect was similar between polyploids (*β′* = 0.28, *P *<* *0.001) and diploids (*β′* = 0.28, *P *<* *0.01). The trait mean of specific leaf area also positively influenced average fitness (Fig. 5a), but the magnitude was stronger in polyploids (*β′* = 0.27, *P *=* *0.015) than in diploids (*β′* = 0.16, *P *=* *0.074). While plants producing foliage of higher vein density and trichome density were associated with lower fitness (Fig. 5a), these negative effects were especially strong in diploids (*β′* = −0.25, *P *<* *0.001, and *β′* = −0.30, *P *<* *0.001, respectively) relative to polyploids (*β′* = −0.08, *P *=* *0.40, and *β′* = −0.10, *P *=* *0.42, respectively).

Trait plasticities had significant positive effects on average fitness for only two of the seven traits (i.e. stomatal length and trichome density; Fig. 5b; Table S8), and the effect sizes for these adaptive plasticities were nearly twofold higher in polyploids (*β′* = 0.28 and 0.29, respectively) than in diploids (0.17 and 0.15, respectively). It is also notable that plasticity in stomatal density was maladaptive for diploids (*β′* = −0.13, *P *=* *0.019) but marginally adaptive for polyploids (*β′* = 0.16, *P *=* *0.084), despite the overall neutral effect on average fitness (Fig. 5b).

## Discussion

Using a worldwide genotype collection of *Fragaria* grown in three different climatic regions (cool/coastal, temperate/valley, arid/montane), we derive important insights into the mechanisms underlying polyploid adaptation to heterogeneous environments. Our results indicate divergence between allopolyploids and diploids in several leaf functional traits. Although different functional traits display varying degrees of plasticity, trait plasticity is of similar magnitude between diploids and allopolyploids, suggesting that increased genomic redundancy does not necessarily translate into greater trait plasticity in polyploids, as is often predicted (Stebbins, 1971; Levin, 1983). More importantly, this is the first study, to our knowledge, to explicitly link functional traits and plasticity to fitness differences between wild polyploids and diploids in natural environments. We find that both trait mean and trait plasticity contribute to higher allopolyploid fitness, and provide support for the ‘jack‐and‐master’ hypothesis for allopolyploid advantage over diploids in the genus *Fragaria*.

### Similar trait plasticity between diploids and polyploids

Our findings of similar trait plasticity between diploids and allopolyploids contradict the long‐held idea (Stebbins, 1971; Levin, 1983) that greater trait plasticity in polyploids enables them to occupy broader ecological niches by expressing suitable phenotypes across a wider range of environments than is the case with diploids. Despite rich theory (Ramsey & Ramsey, 2014), there have been few empirical evaluations of trait plasticity and polyploidy, and none as extensive as our study in terms of the geographic, genetic and phylogenetic diversity of the source material. A glasshouse experiment of trait plasticity in response to water variation (Manzaneda *et al*., 2015) revealed similar plasticity between annual allotetraploid *Brachypodium hybridum* and its diploid progenitor *B. distachyon* in stomatal conductance and carbon isotope discrimination, although the diploid exhibited higher plasticity in photosynthetic rate. In response to nutrient variation (Sánchez Vilas & Pannell, 2017), similar plasticity in specific leaf area was found in glasshouse conditions between autotetraploid and allohexaploid cytotypes of the annual *Mercurialis annua*. For perennial allotetraploid and diploid *Centaurea stoebe* (Hahn *et al*., 2012), similar plasticity between ploidy levels was observed in all measured functional traits in response to water and nutrient variation in garden settings, and only a few traits exhibited higher plasticity in the polyploid in response to garden sites for one of two measuring occasions. These case studies, along with ours, draw the general picture of comparable degrees of plasticity in functional traits between diploids and polyploids. This pattern appears consistent across diverse plant genera, life‐history strategies and environments, suggesting that it may well be the rule rather than the exception, at least for herbaceous polyploid plants.

There are several potential explanations for the lack of differentiation in trait plasticity between ploidy levels. First, polyploidy‐induced versatility in gene and the resultant trait expression (e.g. Gaeta *et al*., 2007) may quickly diminish during the course of polyploid formation, as a result of gene loss or silencing of duplicated copies (Wendel *et al*., 2018), particularly for genes involved in essential biological processes such as photosynthesis (De Smet *et al*., 2013). Second, even given gene retention in polyploids, it is possible that only one copy responds to a specific aspect of the abiotic environment, such as in allotetraploid cotton (Liu & Adams, 2007), where one copy of the alcohol dehydrogenase gene responds to cold stress and the other to water treatment owing to subfunctionalization of gene duplicates. Third, similar trait plasticity between ploidy levels may arise from biased gene expression towards one of the subgenomes in allopolyploids. Such subgenome expression bias has been seen in both synthetic and wild polyploids (Jackson & Chen, 2010; Grover *et al*., 2012), such as *Brassica rapa* (Cheng *et al*., 2016) and *Mimulus peregrinus* (Edger *et al*., 2017). Thus, linking gene expression of polyploids and diploids to trait plasticity will be critical in disentangling the mechanisms underlying similar trait plasticity between ploidy levels, as well as resolving when plasticity in gene expression (Adams & Wendel, 2005; Leitch & Leitch, 2008) is – or is not – correlated with trait plasticity.

### Polyploid fitness advantage and its ecological mechanisms

Among the heterogeneous environments provided by our climatic gardens, allopolyploid *Fragaria* displayed the ‘jack‐and‐master’ strategy, showing higher fitness in each environment, and overall higher average fitness than diploids. Such polyploid fitness advantage has also been detected in the autotetraploids *Arrhenatherum elatius* (Petit & Thompson, 1997) and *Dactylis glomerata* (Bretagnolle & Thompson, 2001), and the allotetraploid *Centaurea stoebe* (Hahn *et al*., 2012). In a *Claytonia* complex (two 2*x*, one 4*x* and two 6*x* cytotypes) growing in California, one 6*x* cytotype possessed higher biomass than the others consistently across elevational gardens, albeit not for polyploid cytotypes as a whole (McIntyre & Strauss, 2017). In our study, although sexual fitness (e.g. flower number and/or fruit production) was not measured, owing to extremely low incidence of flowering across gardens (*c*. 20 plants out of 3137 total), sexual reproduction scales with clonal reproduction in long‐lived, perennial *Fragaria* (Ashman, 2005), unlike in annual plants. Thus, we expect that polyploid advantage also holds when considering sexual fitness in *Fragaria*.

Here allopolyploids performed as habitat generalists relative to diploids; yet, as they did not exhibit fitness homeostasis across climatic gardens, the ‘jack‐of‐all‐trades’ hypothesis must be rejected. Such plasticity in fitness (i.e. enhanced fitness in response to a favorable environment) is ubiquitous in both polyploids and diploids (Petit & Thompson, 1997; Bretagnolle & Thompson, 2001; Buggs & Pannell, 2007; Hahn *et al*., 2012; Sánchez Vilas & Pannell, 2017). Although these previous studies and ours often support the ‘jack‐and‐master’ hypothesis for polyploids (but see Buggs & Pannell, 2007), we cannot rule out the possibility that some diploid *Fragaria* may exhibit the ‘master‐of‐some’ strategy in environments beyond the climatic variation captured by this study (e.g. locations with higher dry season precipitation or greater seasonality; Fig. S3), albeit our gardens are contained within the climatic niches of *Fragaria* species, and niche distances were taken into account. Thus, generalizing about the adaptive strategies of polyploids and diploids will require not only genetically and geographically broad sampling of taxa as we have here, but also more diverse field environments.

Our study is the first to explicitly connect fitness differences between wild polyploids and diploids in natural environments to functional traits and trait plasticity. While trait plasticity contributes to fitness of diploid and allotetraploid *Centaurea stoebe* (Hahn *et al*., 2012), our results revealed not only the importance of trait plasticity for fitness, but also stronger consequences of adaptive plasticity in allopolyploids than in diploids. Relative to trait plasticity, we found that functional trait divergence between allopolyploids and diploids, owing to genomic changes in size and structure (Levin, 1983; Balao *et al*., 2011), probably plays a more important role in determining fitness differences between ploidy levels, as more traits predict fitness in terms of trait means than plasticities. Also significant is the fact that allopolyploids benefit from stronger positive fitness effects and weaker negative fitness effects of their functional traits, perhaps because their trait means are closer to optima than is the case with diploids in the experimental habitats. One should note, however, that these conclusions rest on the assumption that the statistical covariance between traits and fitness reflect causal relationships that would need to be verified with experiments manipulating the predicted causal traits and/or environments (Kingsolver *et al*., 2012).

In conclusion, the broad phylogenetic, genetic and geographic scope of this study provides the most robust evaluation to date of adaptive hypotheses for fitness advantage of wild polyploids in changing environments, and elucidates functional trait divergence and adaptive plasticity as the underlying ecological mechanisms. We emphasize that our findings are based on naturally occurring diploids and allopolyploids; as such, they reflect the ‘effective’ adaptions of allopolyploidy, resulting from the cumulative effects of allopolyploid formation via genome duplication and hybridization, and allopolyploidy‐enabled establishment and divergence. Thus the generalizability of our findings to autopolyploidy (i.e. genome duplication without hybridization) remains to be determined. Such comparisons among diploids, autopolyploids and allopolyploids will ultimately be valuable for informing the respective roles of genome duplication and hybridization on polyploid adaptation. Here in light of allopolyploid fitness advantage, the coexistence of allopolyploid and diploid *Fragaria* in parts of their ranges (Fig. 1) may reflect the collective roles of ecological adaptation to abiotic environment as addressed here as well as other mechanisms (e.g. demographic history, dispersal, and biotic interactions). Future research on linking the biotic adaptation of polyploidy to functional traits and trait plasticity is necessary. Nevertheless, our results add significantly to the understudied ecological adaptations of polyploids, especially allopolyploids (Ramsey & Ramsey, 2014), and offer important insights into the causes of evolutionary success of repeated and pervasive occurrence of polyploids (Van de Peer *et al*., 2017).

## Author contributions

T‐LA and AL designed the research, and all authors contributed to refinement of the design. RC managed garden establishment. All authors collected data. NW performed data analyses. NW wrote the manuscript, and all authors contributed substantially to revisions.

## Supporting information

Please note: Wiley Blackwell are not responsible for the content or functionality of any Supporting Information supplied by the authors. Any queries (other than missing material) should be directed to the *New Phytologist* Central Office.

**Fig. S1** Distinct separation between diploid and high‐order polyploid *Fragaria*, with stomatal length as an example.
**Fig. S2** Collection map of *Fragaria*.
**Fig. S3** Climatic niche distances of 72 source *Fragaria* populations to the common gardens.
**Fig. S4** Maximum likelihood (ML) plastid phylogeny of *Fragaria*.
**Fig. S5** Model comparisons for evolutionary dependence control, with stomatal length as an example.
**Fig. S6** Model comparisons for evolutionary dependence control, with fitness as an example.
**Fig. S7** Central leaflet width was similar between diploid and polyploid *Fragaria* taxa.
**Fig. S8** Similar scales of three fitness components.
**Methods S1** Additional details of Materials and Methods.
**Table S1** Genotypes and populations of diploid and polyploid *Fragaria*.
**Table S2** Soil properties of the three common gardens.
**Table S3** Pairwise correlations between trait means and trait plasticities.
**Table S4** Differences in leaf functional traits between diploids and polyploids.
**Table S5** Pairwise correlations between trait plasticities and between functional traits.
**Table S6** Differences in trait plasticity between diploids and polyploids.
**Table S7** Differences in fitness between diploids and polyploids.
**Table S8** Relationships between average fitness and trait means and trait plasticities.
