# Supplementary material for: Functional trait divergence and trait plasticity confer polyploid advantage in heterogeneous environments
Source: New Phytol. 2018 Oct 27;221(4):2286–97. doi: 10.1111/nph.15508 (PMC6587808; doi:10.1111/nph.15508)
Supplement: Supplementary file 1 — Fig. S1 Distinct separation between diploid and high‐order polyploid Fragaria, with stomatal length as an example. Fig. S2 Collection map of Fragaria. Fig. S3 Climatic niche distances of 72 source Fragaria populations to the common gardens. Fig. S4 Maximum likelihood (ML) plastid phylogeny of Fragaria. Fig. S5 Model comparisons for evolutionary dependence control, with stomatal length as an example. Fig. S6 Model comparisons for evolutionary dependence control, with fitness as an example. Fig. S7 Central leaflet width was similar between diploid and polyploid Fragaria taxa. Fig. S8 Similar scales of three fitness components. Methods S1 Additional details of Materials and Methods. Table S1 Genotypes and populations of diploid and polyploid Fragaria. Table S2 Soil properties of the three common gardens. Table S3 Pairwise correlations between trait means and trait plasticities. Table S4 Differences in leaf functional traits between diploids and polyploids. Table S5 Pairwise correlations between trait plasticities and between functional traits. Table S6 Differences in trait plasticity between diploids and polyploids. Table S7 Differences in fitness between diploids and polyploids. Table S8 Relationships between average fitness and trait means and trait plasticities. [file NPH-221-2286-s001.pdf]

***New Phytologist* Supporting Information**

Article title: **Functional trait divergence and trait plasticity confer polyploid advantage in heterogeneous environments**

Authors: Na Wei, Richard Cronn, Aaron Liston, and Tia-Lynn Ashman

Article acceptance date: 24 September 2018

The following Supporting Information is available for this article:

Methods S1 Additional details of Materials and Methods.

Fig. S1 Distinct separation between diploid and high-order polyploid *Fragaria*, with stomatal length as an example.

Fig. S2 Collection map of *Fragaria*.

Fig. S3 Climatic niche distances of 72 source *Fragaria* populations to the common gardens.

Fig. S4 Maximum-likelihood (ML) plastid phylogeny of *Fragaria*.

Fig. S5 Model comparisons for evolutionary dependence control, with stomatal length as an example.

Fig. S6 Model comparisons for evolutionary dependence control, with fitness as an example.

Fig. S7 Central leaflet width was similar among diploid and polyploid *Fragaria* taxa.

Fig. S8 Similar scales of three fitness components.

Table S1 Genotypes and populations of diploid and polyploid *Fragaria*

Table S2 Soil properties of the three common gardens

Table S3 Pairwise correlations between trait means and trait plasticities

Table S4 Differences in leaf functional traits between diploids and polyploids

Table S5 Pairwise correlations between trait plasticities and between functional traits

Table S6 Differences in trait plasticity between diploids and polyploids

Table S7 Differences in fitness between diploids and polyploids

Table S8 Relationships between average fitness and trait means and trait plasticities

Methods S1 Additional details of Materials and Methods

Common garden soil

Newport beds used a mixture of equal parts beach sand and Bandon fine sandy loam, a low clay content (5–15%) soil derived from the sandy alluvium of coastal marine terraces. Corvallis beds used Chehalis silt clay loam, a richer soil characterized by higher clay content (typically 35–45%). Bend beds used Lundgren ashy sandy loam, a soil characterized by high volcanic ash, glass, and pumice content.

Common garden weather data

We obtained daily data of temperature and rainfall for the three common gardens from different sources: Newport data from the Hatfield Marine Science Center (http://weather.hmsc.oregonstate.edu/weather/weatherproject/archive/), Corvallis and Bend data from AgriMet (https://www.usbr.gov/pn/agrimet/webagdayread.html). We then calculated the monthly mean temperature, monthly rainfall and monthly growing degree days (i.e. the cumulative heat above 10°C) for each garden location, during the course of the field experiment from October 2015 to mid July 2016.

Measurements of leaf functional traits

We collected the largest, fully expanded leaf from each experimental plant in selected beds in each garden. The leaves were scanned using a CanoScan LiDE 220 (Canon, Melville, NY, USA) with an antiglare styrene sheet. We used ImageJ v1.51a (Schneider *et al.*, 2012) to measure leaf area (LA) and the central leaflet width (CLW).

The seven leaf functional traits included: specific leaf area (SLA), which measures the light-capturing leaf area per unit investment of dry mass (Poorter *et al.*, 2009); leaf nitrogen content (N_{mass}), which influences photosynthetic potential (Wright *et al.*, 2004); stomatal length (SL) and stomatal density (SD) that regulate plant CO_2 intake and water transpiration (Hetherington & Woodward, 2003); minor vein density (VLA) that reflects hydraulic conductance (Sack & Scoffoni, 2013); trichome density (TD), which can protect plants against water loss (Ehleringer & Björkman, 1978; Sletvold & Ågren, 2012); and carbon isotope

discrimination ($\Delta^{13}C$) that indicates plant intrinsic water use efficiency (Farquhar & Richards, 1984).

We obtained four leaf punches (each of 6 mm in diameter) from the middle portion of the central leaflet of the collected trifoliate leaf each sample, avoiding the midvein. Two leaf punches were used for measuring stomatal density and stomatal length of the abaxial and adaxial sides; one leaf punch was for measuring SLA; and one was for measuring trichome density and then vein density. When the central leaflet was not large enough for all trait measurements, we obtained two leaf punches from the central leaflet for stomatal density and stomatal length measurements, and one leaf punch from each of the two lateral leaflets for SLA, and trichome density and vein density, respectively.

For SLA estimation, one leaf punch per sample was stored in 96-well microplates (Thermo Fisher Scientific, Hampton, NH, USA), and dried at 65°C for 24 h. Leaf punches were then weighed using a Cahn C-35 microbalance (Thermo Fisher Scientific; with precision of 0.0001 mg). SLA was calculated using the known punch area divided by punch weight.

For stomatal measurements, we used a vinyl polysiloxane impression method to obtain the abaxial and adaxial stomata from leaf punches. First, we mixed the vinyl polysiloxane impression material (Patterson Dental, Pittsburgh, PA, USA) of the base and catalyst, and put the mixture onto a microscope slide. Two punches per sample were placed immediately onto the mixture, one for each side of the leaf. We placed another microscope slide on the top, and pressed slightly and held two slides together using binder clips. After the mixture dried (*c*. 15 min), the top slide and leaf punches were removed from the mixture using forceps to obtain permanent leaf impression. We applied clear nail polish to the impression and peeled off the impression using clear tapes, and placed it onto a new microscope slide for measuring stomatal density and stomatal length. The abaxial and adaxial stomata were counted using a Leica DM500 microscope (Leica Microsystems, Wetzlar, Germany) under 400× (10 × 40) magnification. Specifically, we counted the total number of stomata within two randomly selected fields of view (FOV) for each side. Stomatal density was calculated as the average number of stomata within a FOV divided by the area of the FOV. We took images of the abaxial and adaxial stomata, and measured the guard cell lengths of up to five stomata of each side and

obtained the average stomatal length. As most *Fragaria* plants only produce stomata on the abaxial side, we only reported abaxial stomatal density and stomatal length.

For trichome density estimation, one leaf punch per sample was stored in 70% ethanol. We counted the number of trichomes on both sides of a leaf punch under a dissecting microscope. If there were no more than 50 trichomes on one side, we counted all the trichomes. If there were >50 trichomes on one side, we counted trichomes within two randomly selected areas (each area = 1.5 mm × 1.5 mm) of a leaf punch. We summed the abaxial and adaxial trichome density for calculating TD. Leaf punches were then returned to 70% ethanol for subsequent vein density measurement.

Vein density here is defined as the total lengths of minor veins per unit leaf area. We only focused on minor veins, as they account for >80% of the total veins of a leaf and are key to leaf hydraulic capacity and photosynthesis (Sack & Scoffoni, 2013). We followed the protocol of Quantifying Leaf Vein Traits (http://prometheuswiki.org/tiki-index.php?page=Quantifying+leaf+vein+traits), using leaf punches stored in 70% ethanol. We took leaf vein images using a Leica DM500 microscope under 40× magnification, and used ImageJ to record the total lengths of minor veins within a 1 mm × 1 mm area.

The remaining leaf tissue after four leaf punches being taken was dried at 65°C for 48 h, and sent to the Cornell Isotope Laboratory for carbon isotope composition ($\delta^{13}C$) and N_{mass} analysis using a Thermo Delta V isotope ratio mass spectrometer and a NC2500 elemental analyzer. Carbon isotope discrimination ($\Delta^{13}C$) was calculated using the following formula (Farquhar & Richards, 1984):

$$\Delta^{13}C = \frac{\delta^{13}C_{air} - \delta^{13}C_{plant}}{1 + \delta^{13}C_{plant} / 1000}$$, ‰, where $\delta^{13}C_{air}$ equals -8‰.

Plastid phylogeny

The chloroplast nucleotide supermatrix (with 64645 characters), composed of the diploid and polyploid *Fragaria* taxa in this study (except *F. chiloensis* ssp. *chiloensis*) and three other diploid *Fragaria* taxa, as well as the outgroup *Dasiphora fruticosa* ssp. *floribunda* (Fig. S4), was kindly provided by M.S. Dillenberger (Oregon State University). We performed phylogenetic inference

using the maximum likelihood (ML) method with the GTR+Γ model in RAxML v.8.0.26 (Stamatakis, 2014). Confidence in node support was determined with 1000 bootstrapping replicates.

Phylogenetic general linear mixed models (PLMMs)

PLMMs were performed to validate our use of nested random effects in LMMs (i.e. populations nested in taxa and taxa in ploidy levels, ploidy/taxon/population; see main text) to control for evolutionary dependence among populations and taxa. Here we used the bifurcating, plastid tree (Fig. S4) for fitting PLMMs, due to the difficulty of accounting for reticulate evolutionary histories among diploid and polyploid taxa (Fig. 1) in PLMMs. Owing to the lack of *F. chiloensis* ssp. *chiloensis* in the plastid tree, we assumed that it had the same evolutionary history as *F. chiloensis* ssp. *pacifica*. We conducted PLMMs using the R package MCMCglmm (Hadfield, 2010), with one functional trait (stomatal length, Fig. S5) and the composite fitness (Fig. S6) as examples.

To evaluate how diploids and polyploids differ in stomatal length, similar to the LMM fitted using restricted maximum likelihood (REML) with the package lme4 (Bates *et al.*, 2015) (see main text; Fig. S5, Model 1), we first fitted the same LMM using the Bayesian method with MCMCglmm (Fig. S5, Model 2), where the random effects included ploidy/taxon/population. Then for PLMMs, we fitted two models that differed from the LMMs (Model 1 and Model 2) only in random effects: one PLMM model considered only phylogenetic covariance among taxa (random effects = phylo; Fig. S5, Model 4); one PLMM model considered both populations nested in taxa and phylogenetic covariance among taxa (random effects = taxon/population + phylo; Fig. S5, Model 3). We performed the same four types of models for modeling fitness (Fig. S6).

To fit MCMCglmm models, we used default priors for predictors (fixed effects) and uninformative priors (V = 1, nu = 0.02) for all random effects and residual variance. Models were run with 200000 total MCMC iterations (burin of 100000, and thinning of 100), and convergence was checked graphically. For Bayesian model comparisons based on the deviance

information criterion (DIC), we used the package MuMIn (Bartoń, 2017). Least-squares means of predictors in MCMCglmm models were estimated using the package lsmeans (Lenth, 2016).

For both the functional trait (Fig. S5) and composite fitness (Fig. S6), LMM Model 2 (the Bayesian version of Model 1) with nested random effects outperformed PLMM Model 4 that only considered phylogenetic relatedness among taxa, but performed as well as PLMM Model 3 that considered both populations nested in taxa and phylogenetic relatedness among taxa.

Fig. S1 Distinct separation between diploid and high-order polyploid *Fragaria*, with stomatal length (SL) as an example. The least-squares mean of SL and 1 SEM are plotted. Significant differences are only observed between diploids ($2n = 2x$) and high-order polyploids ($2n \geq 6x$). The response variable (SL) was power transformed to improve normality in a general linear mixed model, where the fixed effects included central leaflet width + climatic niche distance + garden + ploidy + ploidy:garden + ploidy:climatic niche distance, and the nested random effects included ploidy/taxon/population. Owing to the distinct separation between diploids and high-order polyploids, and the dominance of the 8x taxa and genotypes (Table S1; also smaller SEM relative to the 6*x* and 10*x* here), we defined ploidy level broadly as diploid or polyploid in the main text and all downstream analyses.

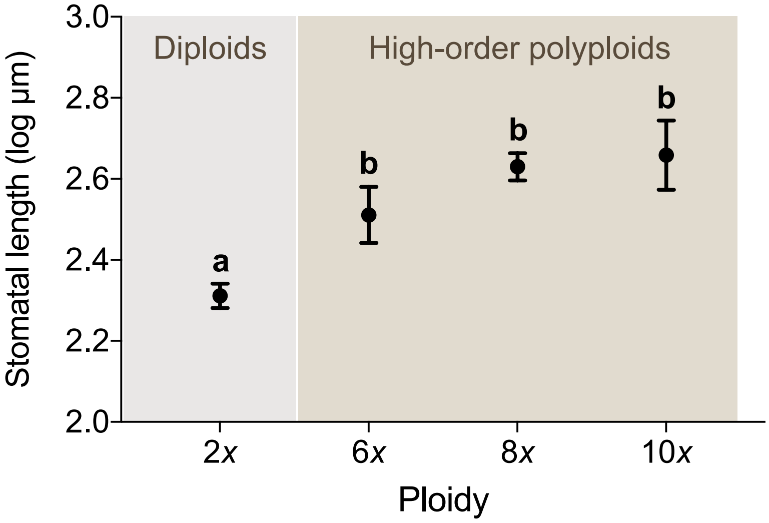

Fig. S2 Collection map of *Fragaria* from our Wild Strawberry website (http://wildstrawberry.org/; accessed on April 25, 2018). This worldwide collection of *Fragaria* was conducted as an international collaborative effort from 2013 to 2014. Each dot represents one population, and the collection data of genotypes within each population are available from the Wild Strawberry website. Briefly, achenes (averagely 70 per plant) were collected from 1–28 plants (mean = 15) of individual populations. For this study, we considered *Fragaria* that occur in North America, South America, Europe and Japan.

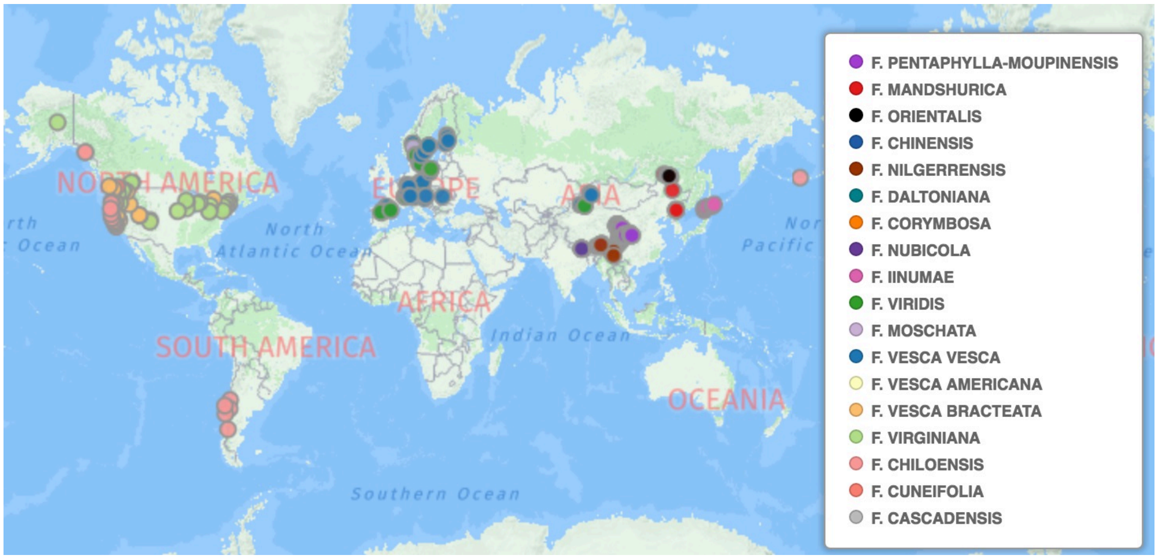

Fig. S3 Climatic niche distances of 72 source *Fragaria* populations to the common gardens. (a) The first two principal components of PCA of the 19 bioclimatic variables and elevation estimates of the 72 source *Fragaria* populations and the three common gardens (the stars). The variables with the largest loadings are indicated by the arrows. (b) The first five PCs, accounting for 94.2% of the variation, were used to calculate Euclidean climatic niche distance between each source population and each garden.

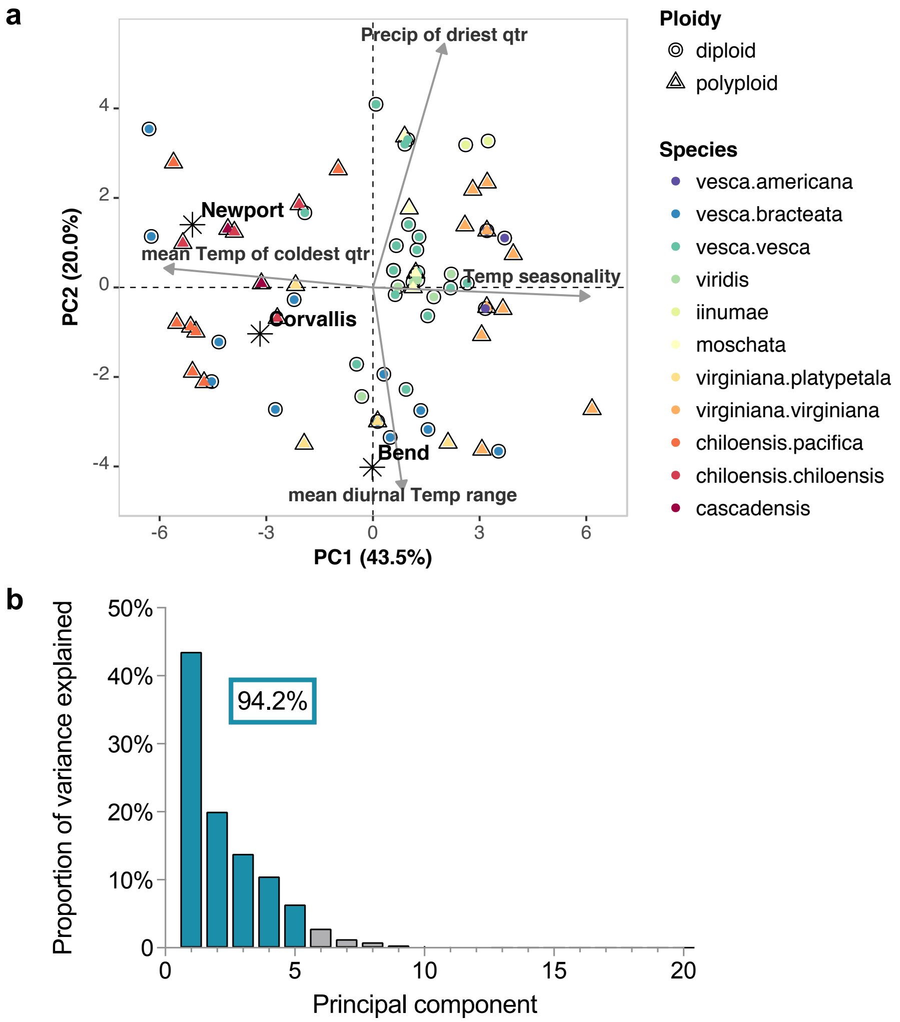

Fig. S4 Maximum-likelihood (ML) plastid phylogeny of *Fragaria*. This phylogeny reflects only the evolutionary histories of the plastid genome, but not the reticulate histories of the nuclear genome among diploid ($2n = 2x$) and polyploid ($2n \geq 6x$) taxa (Fig. 1) that are difficult to be incorporated into general linear mixed models for controlling for evolutionary dependence among taxa. This phylogeny included the diploid and polyploid *Fragaria* in this study (black), and those not (grey). Numbers associated with branches are ML bootstrap support values (%) from 1000 replicates.

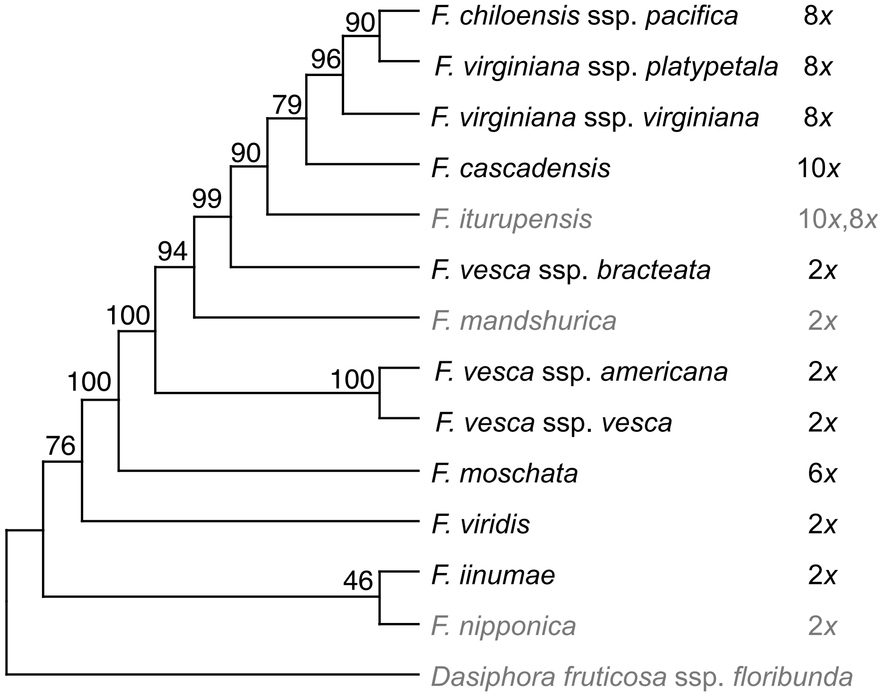

Fig. S5 Model comparisons for controlling for evolutionary dependence among populations and taxa, with stomatal length (SL) as an example. The response variable (SL) was power transformed to improve normality. LMM Model 2 (the Bayesian version of Model 1) with nested random effects outperformed PLMM Model 4 that only considered phylogenetic covariance among taxa. The least-squares mean and 1 SEM are plotted for diploids (blue) and polyploids (red) at each garden location for each model.

Model	Fixed effects	Random effects	Model type	Method	DIC	ΔDIC
1	CLW + climatic niche distance + garden + ploidy + ploidy:garden + ploidy:climatic niche distance	ploidy/taxon/population	LMM	REML[a]		
2		ploidy/taxon/population	LMM	Bayesian[b]	-887.9	0
3		taxon/population + phylo	PLMM	Bayesian[b]	-887.8	0.15
4		phylo	PLMM	Bayesian[b]	-854.1	33.79

LMM, general linear mixed model
PLMM, phylogenetic general linear mixed model
REML, restricted maximum likelihood
[a]using lme4 package; [b]using MCMCglmm package
CLW, central leaflet width

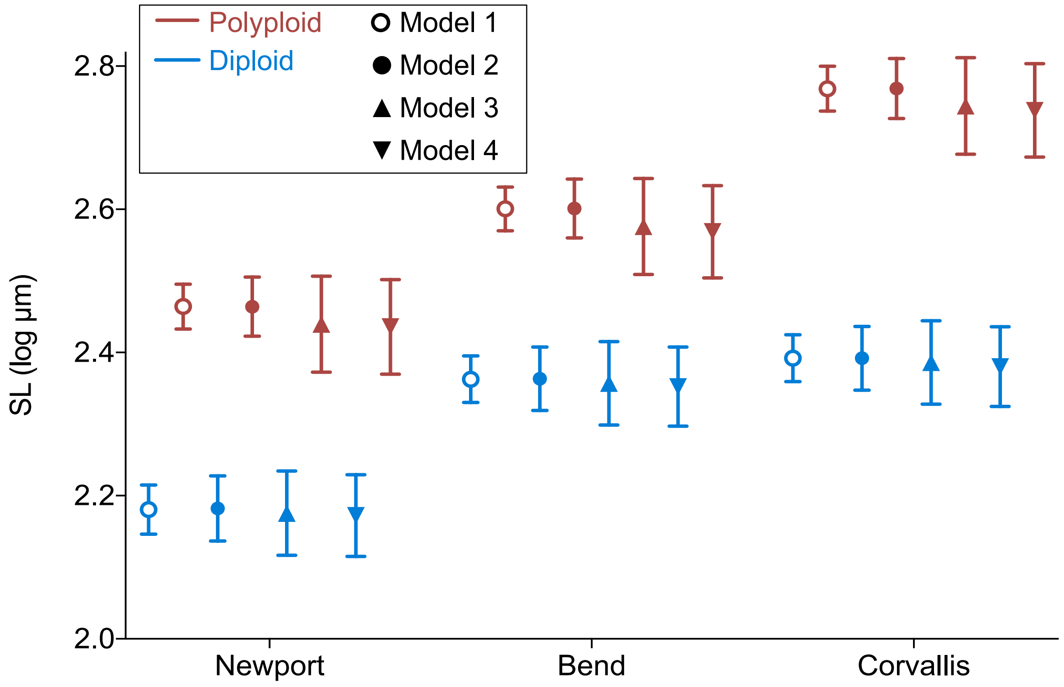

Fig. S6 Model comparisons for controlling for evolutionary dependence among populations and taxa, with the composite fitness as an example. The response variable (the composite fitness index) was power transformed (with power parameter = 0.1) to improve normality. LMM Model 2 (the Bayesian version of Model 1) with nested random effects outperformed PLMM Model 4 that only considered phylogenetic covariance among taxa. The least-squares mean and 1 SEM are plotted for diploids (blue) and polyploids (red) at each garden location for each model.

Model	Fixed effects	Random effects	Model type	Method	DIC	ΔDIC
1	climatic niche distance + garden + ploidy + ploidy:garden + ploidy:climatic niche distance	ploidy/taxon/population	LMM	REML[a]		
2		ploidy/taxon/population	LMM	Bayesian[b]	-426.0	0
3		taxon/population + phylo	PLMM	Bayesian[b]	-424.9	1.1
4		phylo	PLMM	Bayesian[b]	-352.9	73.05

LMM, general linear mixed model
PLMM, phylogenetic general linear mixed model
REML, restricted maximum likelihood
[a]using lme4 package; [b]using MCMCglmm package

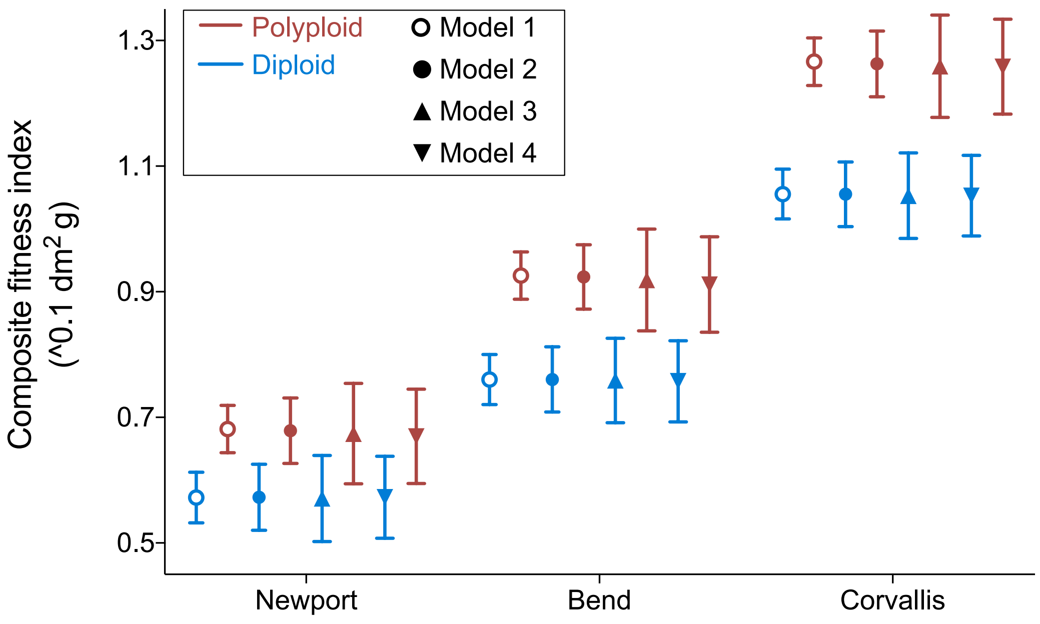

Fig. S7 Central leaflet width was similar among diploid and polyploid *Fragaria* taxa. Individual dots represent genotypic values of each taxon in the three common gardens. The boxes denote the 25th, 50th (median) and 75th percentiles, and whiskers mark 1.5 times the interquartile range from the boxes.

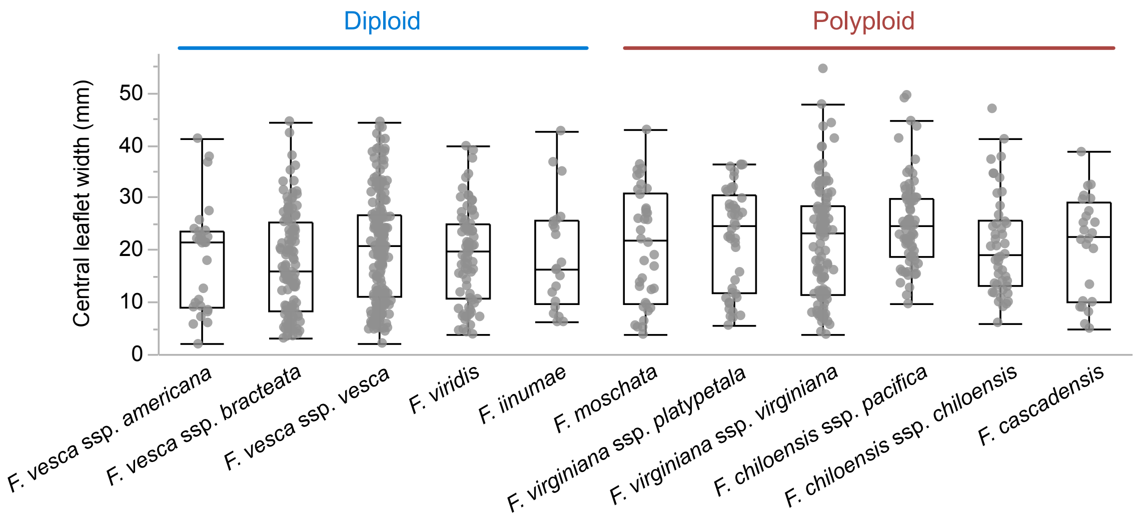

Fig. S8 Similar scales of three fitness components. Genotypic median values of survival rate (median = 1), growth (i.e. plant size since transplanting; median = 0.64 dm^2) and asexual reproduction (i.e. stolon dry mass; 0.56 g), together with the 25th and 75th percentiles, are marked by the boxes. The whiskers mark the range of the 10th and 90th percentiles.

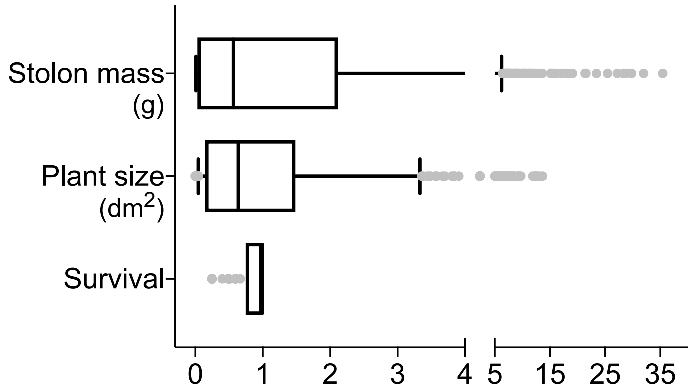

Table S1 Genotypes and populations of diploid and polyploid *Fragaria*

Ploidy	Taxon	Population	Genotype #	Clone #	Latitude	Longitude	Altitude (m)
diploid	*F. vesca ssp. americana*	NA.IA.1	2	23	41.7753	-94.4646	362
diploid	*F. vesca ssp. americana*	NA.NH.3	4	47	44.8710	-71.5036	339
diploid	*F. vesca ssp. americana*	NA.ON.2	4	48	43.4727	-80.0803	307
diploid	*F. vesca ssp. bracteata*	NA.BC.2	1	12	48.7990	-123.1370	188
diploid	*F. vesca ssp. bracteata*	NA.CA.1	4	48	38.7751	-120.4570	1075
diploid	*F. vesca ssp. bracteata*	NA.CA.3	4	48	39.2647	-123.5900	77
diploid	*F. vesca ssp. bracteata*	NA.CA.7	4	48	40.8961	-123.7700	850
diploid	*F. vesca ssp. bracteata*	NA.CO.2	4	48	38.7615	-106.7670	2833
diploid	*F. vesca ssp. bracteata*	NA.ID.1	4	48	44.0270	-115.8550	1146
diploid	*F. vesca ssp. bracteata*	NA.OR.3	4	48	44.4348	-120.3370	1573
diploid	*F. vesca ssp. bracteata*	NA.OR.4	4	48	44.4955	-123.5450	763
diploid	*F. vesca ssp. bracteata*	NA.OR.8	4	48	42.5768	-124.3900	130
diploid	*F. vesca ssp. bracteata*	NA.UT.3	4	48	40.4349	-111.6310	2313
diploid	*F. vesca ssp. bracteata*	NA.WA.2	4	41	48.4819	-118.7270	629
diploid	*F. vesca ssp. bracteata*	NA.WA.3	4	48	47.9640	-117.1010	912
diploid	*F. vesca ssp. vesca*	EU.AT.1	3	36	47.8114	13.0867	558
diploid	*F. vesca ssp. vesca*	EU.CH.5	4	48	46.4909	6.8235	915
diploid	*F. vesca ssp. vesca*	EU.CZ.1	4	48	50.1550	12.2186	632
diploid	*F. vesca ssp. vesca*	EU.DE.5	4	47	47.8636	7.8543	833
diploid	*F. vesca ssp. vesca*	EU.ES.1	4	48	41.2292	-3.4214	1498
diploid	*F. vesca ssp. vesca*	EU.ES.5	4	48	42.0957	0.6254	1051
diploid	*F. vesca ssp. vesca*	EU.FI.4	4	48	62.2333	25.7000	113
diploid	*F. vesca ssp. vesca*	EU.FR.3	4	48	43.3051	-1.2410	242
diploid	*F. vesca ssp. vesca*	EU.HR.1	4	48	45.8680	15.8462	128
diploid	*F. vesca ssp. vesca*	EU.IT.1	4	48	46.1636	10.9217	2100
diploid	*F. vesca ssp. vesca*	EU.NO.1	4	48	60.4119	10.5330	240
diploid	*F. vesca ssp. vesca*	EU.PL.3	4	48	54.2795	18.0036	178
diploid	*F. vesca ssp. vesca*	EU.RO.1	4	48	46.4367	23.7638	347
diploid	*F. vesca ssp. vesca*	EU.SE.1	4	42	55.5222	14.0158	40
diploid	*F. vesca ssp. vesca*	EU.SE.5	4	48	57.7889	11.8332	13
diploid	*F. vesca ssp. vesca*	EU.SI.2	4	48	46.5769	15.6092	332
diploid	*F. viridis*	EU.AT.5	1	12	48.2331	14.8897	230
diploid	*F. viridis*	EU.CZ.4	4	48	50.5508	14.3697	320
diploid	*F. viridis*	EU.CZ.7	4	48	50.4067	13.8067	294
diploid	*F. viridis*	EU.DE.2	4	48	49.8042	7.7410	183
diploid	*F. viridis*	EU.ES.2	4	48	41.4269	-3.7666	1169
diploid	*F. viridis*	EU.NO.3	4	48	60.4332	10.4990	211

diploid	*F. viridis*	EU.SE.1	4	48	55.5222	14.0158	40
diploid	*F. viridis*	EU.SE.10	1	12	59.9255	17.6264	21
diploid	*F. iinumae*	JP.HK.3	4	48	42.8477	141.0960	922
diploid	*F. iinumae*	JP.HK.7	3	36	42.8685	140.6760	795
polyploid	*F. moschata*	EU.AT.4	4	45	47.8125	13.0989	672
polyploid	*F. moschata*	EU.CZ.6	1	12	50.5200	14.3625	285
polyploid	*F. moschata*	EU.SI.1	4	48	46.6827	16.2951	213
polyploid	*F. moschata*	EU.SI.3	4	48	46.2847	15.5876	626
polyploid	*F. virginiana ssp. platypetala*	NA.CA.12	3	36	40.1418	-121.2670	1323
polyploid	*F. virginiana ssp. platypetala*	NA.OR.3	4	48	44.4348	-120.3370	1573
polyploid	*F. virginiana ssp. platypetala*	NA.UT.2	4	48	40.3149	-111.2590	2434
polyploid	*F. virginiana ssp. platypetala*	NA.WA.1	4	47	47.5269	-121.0790	1022
polyploid	*F. virginiana ssp. virginiana*	NA.AB.2	4	47	50.6129	-115.1200	1697
polyploid	*F. virginiana ssp. virginiana*	NA.AK.4	4	48	64.7293	-148.1640	120
polyploid	*F. virginiana ssp. virginiana*	NA.CO.1	4	48	38.1133	-106.9320	3041
polyploid	*F. virginiana ssp. virginiana*	NA.MI.2	4	48	44.6271	-84.5132	349
polyploid	*F. virginiana ssp. virginiana*	NA.NY.1	4	48	41.8640	-74.3461	384
polyploid	*F. virginiana ssp. virginiana*	NA.ON.1	4	48	45.5701	-78.4340	403
polyploid	*F. virginiana ssp. virginiana*	NA.ON.2	4	48	43.4727	-80.0803	307
polyploid	*F. virginiana ssp. virginiana*	NA.PA.1	4	48	41.6415	-80.4329	310
polyploid	*F. virginiana ssp. virginiana*	NA.VT.1	4	48	42.8852	-73.1156	417
polyploid	*F. virginiana ssp. virginiana*	NA.WI.1	2	24	45.2327	-90.6861	392
polyploid	*F. chiloensis ssp. pacifica*	NA.AK.1	4	39	58.4290	-135.7610	21
polyploid	*F. chiloensis ssp. pacifica*	NA.CA.10	4	39	38.3139	-123.0470	4
polyploid	*F. chiloensis ssp. pacifica*	NA.CA.11	3	22	36.3305	-121.8920	38
polyploid	*F. chiloensis ssp. pacifica*	NA.CA.2	5	49	37.4666	-122.4450	11
polyploid	*F. chiloensis ssp. pacifica*	NA.CA.8	4	45	39.4616	-123.8070	18
polyploid	*F. chiloensis ssp. pacifica*	NA.CA.9	4	34	40.7730	-124.2140	3
polyploid	*F. chiloensis ssp. pacifica*	NA.OR.1	4	38	44.9167	-124.0270	5
polyploid	*F. chiloensis ssp. chiloensis*	SA.CL.2	4	48	-45.5500	-72.0667	268
polyploid	*F. chiloensis ssp. chiloensis*	SA.CL.3	4	48	-37.6333	-73.4333	162
polyploid	*F. chiloensis ssp. chiloensis*	SA.CL.4	4	48	-38.7333	-71.2500	1255
polyploid	*F. chiloensis ssp. chiloensis*	SA.CL.5	4	48	-40.5333	-73.2333	11
polyploid	*F. cascadensis*	NA.OR.5	4	48	44.4036	-122.0760	1080
polyploid	*F. cascadensis*	NA.OR.7	4	48	44.5779	-122.1230	1267
**Sum**			**269**	**3137**			

Table S2 Soil properties of the three common gardens

Unit	Variable	Garden: Newport	Corvallis	Bend	Method
%	Sand	89	59	65	Hydrometer method
%	Silt	6	32	28	
%	Clay	5	9	7	
	Moisture	0.4	1.2	0.6	θg
%	C	0.93	0.59	0.59	Elementar
	N	0.07	0.09	0.07	
ratio	C:N	13.3	6.6	8.4	
ppm = mg nutrient/kg soil	NO3-N	1.71	5.49	2.87	Lachat
	P	12.1	33.1	29.0	Mehlich 3 Extraction
	K	41	106	449	
	S	530	650	740	
	Ca	241	1873	1651	
	Mg	41	485	386	
	Mn	2.4	39.4	70.4	
	Cu	1.4	4.9	4.1	
	Zn	0.3	2.1	3.0	
	Fe	12.5	25.6	16.0	
	B	0.3	0.3	0.5	
	Na	25.1	43.7	50.6	Ammonium Acetate Extraction
	CEC (Cation exchange capacity)	2.4	13.5	13.2	Ammonium Acetate Extraction
pH	pH	5.69	6.27	7.67	1:1 soil:water
dS/m	EC (electrical conductivity)	0.099	0.238	0.212	

Soils were collected from each garden in June 2016, and were sent to the Central Analytical Laboratory at Oregon State University for analysis.

Table S3 Pairwise correlations between trait means and trait plasticities for diploids and polyploids

Pairwise comparison		All		Diploids		Polyploids	
		Correlation coefficient (*r*)	*P* value	Correlation coefficient (*r*)	*P* value	Correlation coefficient (*r*)	*P* value
SLA.RDPI	SLA.mean	0.08	0.210	-0.02	0.782	0.17	0.075
SD.RDPI	SD.mean	0.03	0.612	-0.01	0.872	0.06	0.499
SL.RDPI	SL.mean	0.10	0.111	-0.15	0.071	0.10	0.285
VLA.RDPI	VLA.mean	0.01	0.864	-0.08	0.372	0.14	0.155
TD.RDPI	TD.mean	-0.17	**0.010**	-0.21	**0.018**	-0.19	0.057
Δ^{13}C.RDPI	Δ^{13}C.mean	-0.02	0.889	0.14	0.408	-0.11	0.573
N_{mass}.RDPI	N_{mass}.mean	-0.11	0.363	-0.10	0.549	-0.11	0.572

Non-parametric Kendall rank correlation coefficient (*r*) was estimated using the R package psych (Revelle, 2017). Functional trait mean was genotypic trait value averaged across all garden environments.

Table S4 Differences in leaf functional traits between diploids and polyploids

		ANOVA table with Type III sums of squares			
**Functional trait**	**Fixed effects (Predictors)**	**Sum Sq**	**df**	***F***	**Pr(>*F*)**
SLA	central leaflet width	36.36	1	10.38	0.001
	climatic niche distance	11.00	1	3.14	0.077
	garden	712.47	2	101.66	< 2.2e-16
	ploidy	10.87	1	3.10	0.100
	ploidy:garden	55.94	2	7.98	0.000
	ploidy:climatic niche distance	5.11	1	1.46	0.228
	R^2_m : 0.448				
	R^2_c : 0.750				
SL (log)	central leaflet width	0.001	1	0.06	0.805
	climatic niche distance	0.008	1	0.49	0.486
	garden	3.093	2	91.38	< 2.2e-16
	ploidy	0.636	1	37.56	0.000
	ploidy:garden	0.606	2	17.90	0.000
	ploidy:climatic niche distance	0.036	1	2.13	0.145
	R^2_m : 0.594				
	R^2_c : 0.700				
SD (sqrt)	central leaflet width	0.37	1	0.12	0.734
	climatic niche distance	14.82	1	4.60	0.032
	garden	83.61	2	12.96	0.000
	ploidy	13.55	1	4.20	0.049
	ploidy:garden	37.91	2	5.88	0.003
	ploidy:climatic niche distance	3.11	1	0.96	0.327
	R^2_m : 0.105				
	R^2_c : 0.346				
VLA (log)	central leaflet width	0.239	1	8.43	0.004
	climatic niche distance	0.110	1	3.89	0.050
	garden	0.455	1	16.02	0.000
	ploidy	0.135	1	4.74	0.037
	ploidy:garden	0.008	1	0.28	0.597
	ploidy:climatic niche distance	0.035	1	1.25	0.265
	R^2_m : 0.359				
	R^2_c : 0.530				

TD (sqrt)	central leaflet width	2.43	1	10.96	0.001
	climatic niche distance	0.46	1	2.09	0.149
	garden	70.03	1	316.10	< 2.2e-16
	ploidy	0.21	1	0.96	0.346
	ploidy:garden	1.83	1	8.26	0.004
	ploidy:climatic niche distance	0.01	1	0.04	0.840
	R^2_m : 0.249				
	R^2_c : 0.799				
N_{mass}	central leaflet width	0.79	1	4.64	0.033
	climatic niche distance	0.02	1	0.10	0.747
	garden	29.12	2	85.21	< 2e-16
	ploidy	0.10	1	0.57	0.453
	ploidy:garden	1.02	2	2.99	0.053
	ploidy:climatic niche distance	0.15	1	0.88	0.349
	R^2_m : 0.660				
	R^2_c : 0.701				
$\Delta^{13}C$	central leaflet width	1.14	1	2.22	0.138
	climatic niche distance	4.25	1	8.30	0.004
	garden	19.79	2	19.34	0.000
	ploidy	0.75	1	1.46	0.233
	ploidy:garden	1.50	2	1.47	0.234
	ploidy:climatic niche distance	1.10	1	2.15	0.144
	R^2_m : 0.220				
	R^2_c : 0.448				

General linear mixed model (LMM) specification:
model <- lmer(Functional trait ~ Fixed effects + (1 | Nested random effects))
Fixed effects: central leaflet width + climatic niche distance + garden + ploidy + ploidy:garden + ploidy:climatic niche distance
Nested random effects: ploidy/taxon/population
The response variable of each LMM was power transformed if necessary. R^2_m, model marginal R^2 representing variance explained by fixed effects; R^2_c, model conditional R^2 representing variance explained by both fixed effects and random effects.

Table S5 Pairwise correlations between trait plasticities and between functional traits for each taxon

			Trait plasticity RDPI correlation		Trait mean correlation	
**Taxon**	**Trait**	**Trait**	**Correlation coefficient (*r*)**	***P* value**	**Correlation coefficient (*r*)**	***P* value**
Fragaria vesca ssp. *americana*	SLA	SD	-0.11	0.760	-0.24	0.496
	SLA	SL	-0.07	0.855	0.16	0.668
	SD	SL	-0.11	0.760	0.07	0.855
	SLA	VLA	-0.11	0.776	-0.06	0.887
	SD	VLA	0.22	0.566	-0.50	0.170
	SL	VLA	0.39	0.301	-0.28	0.469
	SLA	TD	0.39	0.301	0.33	0.381
	SD	TD	0.17	0.668	-0.44	0.231
	SL	TD	-0.11	0.776	-0.11	0.776
	VLA	TD	0.06	0.887	0.50	0.170
	SLA	$\Delta^{13}C$	-0.33	0.784	0.33	0.784
	SD	$\Delta^{13}C$	-0.33	0.784	-0.33	0.784
	SL	$\Delta^{13}C$	-0.33	0.784	1.00	**0.000**
	VLA	$\Delta^{13}C$	0.33	0.784	-0.33	0.784
	TD	$\Delta^{13}C$	0.33	0.784	-0.33	0.784
	SLA	N_{mass}	-0.33	0.784	1.00	**0.000**
	SD	N_{mass}	1.00	**0.000**	-1.00	**0.000**
	SL	N_{mass}	1.00	**0.000**	0.33	0.784
	VLA	N_{mass}	0.33	0.784	0.33	0.784
	TD	N_{mass}	-1.00	**0.000**	0.33	0.784
	$\Delta^{13}C$	N_{mass}	-0.33	0.784	0.33	0.784
Fragaria vesca ssp. *bracteata*	SLA	SD	-0.02	0.893	-0.10	0.522
	SLA	SL	-0.01	0.972	0.14	0.361
	SD	SL	-0.11	0.484	-0.15	0.337
	SLA	VLA	0.01	0.975	0.15	0.484
	SD	VLA	-0.24	0.248	-0.15	0.484
	SL	VLA	-0.18	0.389	-0.20	0.338
	SLA	TD	-0.05	0.800	0.07	0.728
	SD	TD	0.02	0.924	0.03	0.874
	SL	TD	-0.13	0.525	-0.19	0.354
	VLA	TD	0.07	0.728	0.34	0.096
	SLA	$\Delta^{13}C$	-0.24	0.496	-0.24	0.496
	SD	$\Delta^{13}C$	0.38	0.282	0.24	0.496
	SL	$\Delta^{13}C$	-0.02	0.951	0.07	0.855

	VLA	$\Delta^{13}C$	-0.50	0.170	0.06	0.887
	TD	$\Delta^{13}C$	-0.22	0.566	0.22	0.566
	SLA	N_{mass}	0.56	0.095	0.04	0.902
	SD	N_{mass}	-0.16	0.668	0.31	0.376
	SL	N_{mass}	0.33	0.347	-0.09	0.805
	VLA	N_{mass}	0.28	0.469	-0.33	0.381
	TD	N_{mass}	-0.11	0.776	-0.06	0.887
	$\Delta^{13}C$	N_{mass}	-0.42	0.224	0.27	0.451
Fragaria vesca ssp. *vesca*	SLA	SD	0.02	0.889	0.10	0.429
	SLA	SL	0.06	0.624	-0.03	0.833
	SD	SL	0.08	0.531	-0.07	0.588
	SLA	VLA	-0.11	0.386	0.05	0.687
	SD	VLA	0.06	0.630	0.15	0.226
	SL	VLA	-0.07	0.613	-0.16	0.216
	SLA	TD	0.00	0.978	-0.04	0.741
	SD	TD	0.04	0.783	-0.10	0.429
	SL	TD	0.10	0.441	-0.03	0.833
	VLA	TD	0.11	0.377	0.14	0.282
	SLA	$\Delta^{13}C$	-0.12	0.667	0.37	0.162
	SD	$\Delta^{13}C$	0.10	0.713	-0.05	0.854
	SL	$\Delta^{13}C$	0.25	0.350	0.08	0.759
	VLA	$\Delta^{13}C$	-0.13	0.623	0.18	0.497
	TD	$\Delta^{13}C$	-0.15	0.579	0.02	0.951
	SLA	N_{mass}	0.03	0.902	-0.10	0.713
	SD	N_{mass}	0.22	0.420	0.12	0.667
	SL	N_{mass}	0.13	0.623	-0.08	0.759
	VLA	N_{mass}	0.02	0.951	-0.18	0.497
	TD	N_{mass}	0.30	0.259	0.05	0.854
	$\Delta^{13}C$	N_{mass}	-0.05	0.854	-0.37	0.162
Fragaria viridis	SLA	SD	0.08	0.687	-0.02	0.917
	SLA	SL	-0.08	0.704	0.22	0.284
	SD	SL	-0.21	0.306	0.03	0.870
	SLA	VLA	0.11	0.613	-0.02	0.920
	SD	VLA	-0.01	0.973	-0.03	0.893
	SL	VLA	0.16	0.460	0.04	0.867
	SLA	TD	0.02	0.920	0.05	0.814
	SD	TD	-0.01	0.973	0.00	1.000
	SL	TD	0.32	0.136	-0.17	0.436
	VLA	TD	0.10	0.637	0.14	0.499
	SLA	$\Delta^{13}C$	0.07	0.867	-0.29	0.493
	SD	$\Delta^{13}C$	0.21	0.610	-0.29	0.493
	SL	$\Delta^{13}C$	-0.36	0.385	0.00	1.000

	VLA	$\Delta^{13}C$	-0.07	0.867	0.21	0.610
	TD	$\Delta^{13}C$	-0.07	0.867	0.43	0.289
	SLA	N_{mass}	0.36	0.385	0.50	0.207
	SD	N_{mass}	-0.21	0.610	-0.07	0.867
	SL	N_{mass}	0.36	0.385	0.36	0.385
	VLA	N_{mass}	-0.07	0.867	0.14	0.736
	TD	N_{mass}	0.36	0.385	-0.07	0.867
	$\Delta^{13}C$	N_{mass}	-0.57	0.139	-0.21	0.610
Fragaria iinumae	SLA	SD	0.33	0.465	0.33	0.465
	SLA	SL	-0.05	0.919	-0.52	0.228
	SD	SL	0.24	0.607	-0.81	**0.027**
	SLA	VLA	-0.14	0.760	-0.05	0.919
	SD	VLA	0.14	0.760	0.24	0.607
	SL	VLA	-0.62	0.138	-0.24	0.607
	SLA	TD	-0.24	0.607	0.33	0.465
	SD	TD	0.05	0.919	0.43	0.337
	SL	TD	-0.52	0.228	-0.43	0.337
	VLA	TD	0.52	0.228	-0.33	0.465
	SLA	$\Delta^{13}C$	–	–	–	–
	SD	$\Delta^{13}C$	–	–	–	–
	SL	$\Delta^{13}C$	–	–	–	–
	VLA	$\Delta^{13}C$	–	–	–	–
	TD	$\Delta^{13}C$	–	–	–	–
	SLA	N_{mass}	–	–	–	–
	SD	N_{mass}	–	–	–	–
	SL	N_{mass}	–	–	–	–
	VLA	N_{mass}	–	–	–	–
	TD	N_{mass}	–	–	–	–
	$\Delta^{13}C$	N_{mass}	–	–	–	–
Fragaria moschata	SLA	SD	-0.21	0.501	-0.03	0.934
	SLA	SL	0.05	0.868	-0.10	0.739
	SD	SL	-0.08	0.803	-0.21	0.501
	SLA	VLA	-0.15	0.638	0.06	0.852
	SD	VLA	0.18	0.572	0.21	0.508
	SL	VLA	-0.03	0.926	-0.18	0.572
	SLA	TD	-0.27	0.391	-0.15	0.638
	SD	TD	0.61	**0.037**	-0.06	0.852
	SL	TD	-0.21	0.508	0.21	0.508
	VLA	TD	0.15	0.638	0.55	0.067
	SLA	$\Delta^{13}C$	0.00	1.000	-1.00	0.000
	SD	$\Delta^{13}C$	-0.33	0.667	0.33	0.667
	SL	$\Delta^{13}C$	0.00	1.000	-0.67	0.333

	VLA	$\Delta^{13}C$	0.00	1.000	0.33	0.667
	TD	$\Delta^{13}C$	0.00	1.000	-0.33	0.667
	SLA	N_{mass}	0.00	1.000	0.00	1.000
	SD	N_{mass}	0.33	0.667	0.67	0.333
	SL	N_{mass}	0.67	0.333	-0.33	0.667
	VLA	N_{mass}	0.00	1.000	0.67	0.333
	TD	N_{mass}	0.67	0.333	0.00	1.000
	$\Delta^{13}C$	N_{mass}	0.33	0.667	0.00	1.000
Fragaria virginiana ssp. *platypetala*	SLA	SD	0.01	0.973	-0.10	0.710
	SLA	SL	-0.05	0.866	-0.01	0.973
	SD	SL	0.56	**0.029**	-0.39	0.150
	SLA	VLA	-0.09	0.761	0.16	0.564
	SD	VLA	0.22	0.433	-0.07	0.813
	SL	VLA	0.39	0.150	-0.12	0.660
	SLA	TD	0.12	0.660	-0.01	0.973
	SD	TD	0.05	0.866	0.33	0.225
	SL	TD	-0.16	0.564	-0.22	0.433
	VLA	TD	-0.01	0.973	-0.28	0.319
	SLA	$\Delta^{13}C$	0.00	1.000	0.67	0.333
	SD	$\Delta^{13}C$	0.00	1.000	0.00	1.000
	SL	$\Delta^{13}C$	0.00	1.000	-0.67	0.333
	VLA	$\Delta^{13}C$	0.00	1.000	-1.00	**0.000**
	TD	$\Delta^{13}C$	-0.33	0.667	0.00	1.000
	SLA	N_{mass}	0.00	1.000	0.33	0.667
	SD	N_{mass}	0.00	1.000	-0.33	0.667
	SL	N_{mass}	0.00	1.000	0.33	0.667
	VLA	N_{mass}	0.00	1.000	0.00	1.000
	TD	N_{mass}	0.33	0.667	-1.00	**0.000**
	$\Delta^{13}C$	N_{mass}	0.33	0.667	0.00	1.000
Fragaria virginiana ssp. *virginiana*	SLA	SD	-0.04	0.793	0.21	0.211
	SLA	SL	0.07	0.690	-0.28	0.092
	SD	SL	0.18	0.286	-0.46	**0.003**
	SLA	VLA	-0.02	0.926	0.27	0.106
	SD	VLA	0.02	0.926	0.27	0.097
	SL	VLA	-0.02	0.899	-0.28	0.085
	SLA	TD	0.15	0.380	0.07	0.678
	SD	TD	-0.04	0.819	-0.04	0.799
	SL	TD	0.37	**0.021**	-0.15	0.371
	VLA	TD	0.06	0.728	0.25	0.136
	SLA	$\Delta^{13}C$	-0.38	0.282	0.07	0.855
	SD	$\Delta^{13}C$	0.56	0.095	0.16	0.668
	SL	$\Delta^{13}C$	0.07	0.855	0.24	0.496

	VLA	$\Delta^{13}C$	-0.02	0.951	-0.42	0.224
	TD	$\Delta^{13}C$	-0.20	0.580	-0.07	0.855
	SLA	N_{mass}	0.20	0.580	-0.20	0.580
	SD	N_{mass}	-0.20	0.580	0.07	0.855
	SL	N_{mass}	0.02	0.951	0.16	0.668
	VLA	N_{mass}	-0.16	0.668	-0.33	0.347
	TD	N_{mass}	0.20	0.580	-0.33	0.347
	$\Delta^{13}C$	N_{mass}	-0.02	0.951	0.29	0.418
Fragaria chiloensis ssp. *pacifica*	SLA	SD	0.06	0.754	0.06	0.754
	SLA	SL	0.11	0.601	-0.19	0.343
	SD	SL	-0.08	0.709	-0.27	0.186
	SLA	VLA	0.32	0.140	-0.08	0.716
	SD	VLA	-0.16	0.476	0.11	0.632
	SL	VLA	-0.18	0.429	-0.05	0.833
	SLA	TD	0.07	0.774	-0.28	0.226
	SD	TD	0.07	0.774	-0.29	0.209
	SL	TD	-0.02	0.935	0.24	0.299
	VLA	TD	-0.43	0.053	0.12	0.593
	SLA	$\Delta^{13}C$	0.24	0.607	-0.05	0.919
	SD	$\Delta^{13}C$	0.33	0.465	-0.43	0.337
	SL	$\Delta^{13}C$	-0.33	0.465	-0.24	0.607
	VLA	$\Delta^{13}C$	0.05	0.919	0.43	0.337
	TD	$\Delta^{13}C$	0.24	0.607	0.71	0.071
	SLA	N_{mass}	0.43	0.337	0.24	0.607
	SD	N_{mass}	-0.24	0.607	0.43	0.337
	SL	N_{mass}	-0.14	0.760	0.43	0.337
	VLA	N_{mass}	-0.14	0.760	-0.43	0.337
	TD	N_{mass}	0.43	0.337	-0.33	0.465
	$\Delta^{13}C$	N_{mass}	0.43	0.337	-0.62	0.138
Fragaria chiloensis ssp. *chiloensis*	SLA	SD	-0.10	0.713	-0.20	0.458
	SLA	SL	-0.05	0.854	0.13	0.623
	SD	SL	0.15	0.579	-0.27	0.318
	SLA	VLA	-0.05	0.873	0.24	0.484
	SD	VLA	0.05	0.873	0.02	0.958
	SL	VLA	-0.02	0.958	-0.09	0.790
	SLA	TD	-0.36	0.245	0.42	0.169
	SD	TD	0.27	0.391	-0.09	0.779
	SL	TD	0.42	0.169	-0.09	0.779
	VLA	TD	-0.16	0.631	0.45	0.160
	SLA	$\Delta^{13}C$	-0.33	0.667	0.33	0.667
	SD	$\Delta^{13}C$	0.67	0.333	-1.00	**0.000**
	SL	$\Delta^{13}C$	-0.33	0.667	-0.67	0.333

	VLA	$\Delta^{13}C$	-0.33	0.784	-0.33	0.784
	TD	$\Delta^{13}C$	0.33	0.667	0.67	0.333
	SLA	N_{mass}	-0.33	0.667	0.67	0.333
	SD	N_{mass}	-0.67	0.333	-0.67	0.333
	SL	N_{mass}	1.00	**0.000**	-0.33	0.667
	VLA	N_{mass}	1.00	**0.000**	-0.33	0.784
	TD	N_{mass}	0.33	0.667	1.00	**0.000**
	$\Delta^{13}C$	N_{mass}	-0.33	0.667	0.67	0.333
Fragaria cascadensis	SLA	SD	0.21	0.610	-0.36	0.385
	SLA	SL	0.29	0.493	0.00	1.000
	SD	SL	0.07	0.867	-0.50	0.207
	SLA	VLA	-0.64	0.086	0.50	0.207
	SD	VLA	0.00	1.000	-0.14	0.736
	SL	VLA	-0.36	0.385	-0.36	0.385
	SLA	TD	-0.07	0.867	-0.14	0.736
	SD	TD	0.00	1.000	0.36	0.385
	SL	TD	-0.07	0.867	-0.29	0.493
	VLA	TD	-0.14	0.736	0.07	0.867
	SLA	$\Delta^{13}C$	–	–	–	–
	SD	$\Delta^{13}C$	–	–	–	–
	SL	$\Delta^{13}C$	–	–	–	–
	VLA	$\Delta^{13}C$	–	–	–	–
	TD	$\Delta^{13}C$	–	–	–	–
	SLA	N_{mass}	–	–	–	–
	SD	N_{mass}	–	–	–	–
	SL	N_{mass}	–	–	–	–
	VLA	N_{mass}	–	–	–	–
	TD	N_{mass}	–	–	–	–
	$\Delta^{13}C$	N_{mass}	–	–	–	–

Non-parametric Kendall rank correlation coefficient (r) was estimated using the R package psych (Revelle, 2017). Functional trait mean was genotypic trait value averaged across all garden environments. Missing r values were due to few data for carbon isotope discrimination and nitrogen content in some taxa.

Table S6 Differences in trait plasticity between diploids and polyploids

Plasticity index	Trait plasticity	Fixed effects (Predictors)	ANOVA table with Type III sums of squares			
			**Sum Sq**	**df**	***F***	**Pr(>F)**
RDPI	SLA.RDPI	climatic niche distance mean	0.0024	1	1.00	0.323
		ploidy	0.0022	1	0.89	0.353
		ploidy:climatic niche distance mean	0.0021	1	0.86	0.358
		R^2_m : 0.012				
		R^2_c : 0.154				
	SL.RDPI	climatic niche distance mean	0.0038	1	1.75	0.200
		ploidy	0.0035	1	1.62	0.221
		ploidy:climatic niche distance mean	0.0012	1	0.56	0.462
		R^2_m : 0.051				
		R^2_c : 0.173				
	SD.RDPI (sqrt)	climatic niche distance mean	0.0008	1	0.06	0.801
		ploidy	0.0045	1	0.37	0.547
		ploidy:climatic niche distance mean	0.0153	1	1.25	0.273
		R^2_m : 0.026				
		R^2_c : 0.088				
	VLA.RDPI (sqrt)	climatic niche distance mean	0.0170	1	1.08	0.305
		ploidy	0.0001	1	0.01	0.934
		ploidy:climatic niche distance mean	0.0002	1	0.01	0.920
		R^2_m : 0.006				
		R^2_c : 0.084				
	TD.RDPI	climatic niche distance mean	0.0009	1	0.03	0.865
		ploidy	0.0653	1	2.14	0.150
		ploidy:climatic niche distance mean	0.0945	1	3.10	0.084
		R^2_m : 0.015				
		R^2_c : 0.327				
	N_{mass}.RDPI	climatic niche distance mean	0.0358	1	5.51	0.029
		ploidy	0.0001	1	0.02	0.881
		ploidy:climatic niche distance mean	0.0002	1	0.03	0.872
		R^2_m : 0.082				
		R^2_c : 0.109				

	$\Delta^{13}C$.RDPI (sqrt)	climatic niche distance mean	0.0003	1	0.14	0.714
		ploidy	0.0011	1	0.58	0.459
		ploidy:climatic niche distance mean	0.0001	1	0.06	0.808
		R^2_m : 0.077				
		R^2_c : 0.083				
PI	SLA.PI	climatic niche distance mean	0.0024	1	0.41	0.526
		ploidy	0.0042	1	0.71	0.406
		ploidy:climatic niche distance mean	0.0038	1	0.64	0.427
		R^2_m : 0.007				
		R^2_c : 0.150				
	SL.PI	climatic niche distance mean	0.0109	1	1.81	0.192
		ploidy	0.0103	1	1.70	0.207
		ploidy:climatic niche distance mean	0.0042	1	0.70	0.411
		R^2_m : 0.043				
		R^2_c : 0.135				
	SD.PI (sqrt)	climatic niche distance mean	0.0085	1	0.36	0.551
		ploidy	0.0197	1	0.83	0.365
		ploidy:climatic niche distance mean	0.0358	1	1.49	0.223
		R^2_m : 0.012				
		R^2_c : 0.012				
	VLA.PI (sqrt)	climatic niche distance mean	0.0364	1	1.00	0.323
		ploidy	0.0000	1	0.00	0.982
		ploidy:climatic niche distance mean	0.0000	1	0.00	0.979
		R^2_m : 0.006				
		R^2_c : 0.086				
	TD.PI	climatic niche distance mean	0.0001	1	0.00	0.968
		ploidy	0.1507	1	3.09	0.084
		ploidy:climatic niche distance mean	0.1595	1	3.27	0.075
		R^2_m : 0.017				
		R^2_c : 0.060				
	N_{mass}.PI	climatic niche distance mean	0.0709	1	6.25	0.022
		ploidy	0.0001	1	0.01	0.919
		ploidy:climatic niche distance mean	0.0001	1	0.01	0.932
		R^2_m : 0.087				
		R^2_c : 0.093				

$\Delta^{13}C$.PI (sqrt)	climatic niche distance mean	0.0003	1	0.09	0.772
	ploidy	0.0027	1	0.72	0.409
	ploidy:climatic niche distance mean	0.0005	1	0.12	0.735
	R^2_m : 0.078				
	R^2_c : 0.093				

General linear mixed model (LMM) specification:
model <- lmer(Trait plasticity ~ Fixed effects + (1 | Nested random effects))
Fixed effects: climatic niche distance mean (i.e. genotypic climatic niche distance averaged across all garden environments) + ploidy + ploidy:climatic niche distance mean
Nested random effects: ploidy/taxon/population
RDPI, relative distance plasticity index; PI phenotypic plasticity index. The response variable of each LMM was power transformed if necessary. R^2_m, model marginal R^2 representing variance explained by fixed effects; R^2_c, model conditional R^2 representing variance explained by both fixed effects and random effects.

Table S7 Differences in fitness between diploids and polyploids

Fitness	Fixed effects (Predictors)	ANOVA table with Type III sums of squares			
		Sum Sq	df	F	Pr(>F)
(Composite fitness index)$^{0.1}$	climatic niche distance	2.23	1	71.54	2.22E-16
	garden	35.08	2	562.96	< 2.2E-16
	ploidy	0.62	1	20.02	7.05E-05
	ploidy:garden	0.31	2	4.99	0.007
	ploidy:climatic niche distance	0.32	1	10.24	0.001
	R^2_m : 0.614				
	R^2_c : 0.712				

General linear mixed model (LMM) specification:
model <- lmer(Fitness ~ Fixed effects + (1 | Nested random effects))
Fixed effects: climatic niche distance + garden + ploidy + ploidy:garden + ploidy:climatic niche distance
Nested random effects: ploidy/taxon/population
The response variable of the LMM was power transformed (power parameter = 0.1). R^2_m, model marginal R^2 representing variance explained by fixed effects; R^2_c, model conditional R^2 representing variance explained by both fixed effects and random effects.

Table S8 Relationships between average fitness and trait means and trait plasticities in heterogeneous garden environments

Functional trait for model	Fixed effects (Predictors)	ANOVA table with Type III sums of squares			
		**Sum Sq**	**df**	***F***	**Pr(>*F*)**
SLA	climatic niche distance mean	0.055	1	2.22	0.141
	SLA.RDPI	0.034	1	1.37	0.243
	SLA.mean	0.137	1	5.57	**0.019**
	ploidy:SLA.RDPI	0.004	1	0.17	0.681
	ploidy:SLA.mean	0.049	1	1.99	0.175
	R^2_m : 0.053				
	R^2_c : 0.581				
SL	climatic niche distance mean	0.043	1	1.91	0.172
	SL.RDPI	0.511	1	22.72	**0.000**
	SL.mean	0.235	1	10.46	**0.001**
	ploidy:SL.RDPI	0.033	1	1.47	0.226
	ploidy:SL.mean	0.000	1	0.01	0.925
	R^2_m : 0.253				
	R^2_c : 0.553				
SD	climatic niche distance mean	0.049	1	2.01	0.161
	SD.RDPI	0.001	1	0.05	0.818
	SD.mean	0.013	1	0.55	0.459
	ploidy:SD.RDPI	0.185	1	7.58	**0.006**
	ploidy:SD.mean	0.030	1	1.23	0.276
	R^2_m : 0.192				
	R^2_c : 0.539				
VLA	climatic niche distance mean	0.008	1	0.36	0.553
	VLA.RDPI	0.000	1	0.00	0.997
	VLA.mean	0.106	1	4.69	**0.031**
	ploidy:VLA.RDPI	0.085	1	3.76	0.054
	ploidy:VLA.mean	0.159	1	7.03	**0.015**
	R^2_m : 0.203				
	R^2_c : 0.575				
TD	climatic niche distance mean	0.020	1	0.98	0.326
	TD.RDPI	0.360	1	17.62	**0.000**
	TD.mean	0.122	1	5.98	**0.015**
	ploidy:TD.RDPI	0.048	1	2.36	0.126
	ploidy:TD.mean	0.050	1	2.45	0.124
	R^2_m : 0.265				

	R^2_c : 0.662				
N_{mass}	climatic niche distance mean	0.020	1	0.93	0.340
	N_{mass}.RDPI	0.000	1	0.00	0.969
	N_{mass}.mean	0.000	1	0.01	0.927
	ploidy:N_{mass}.RDPI	0.000	1	0.00	0.948
	ploidy:N_{mass}.mean	0.066	1	3.06	0.088
	R^2_m : 0.227				
	R^2_c : 0.547				
$\Delta^{13}C$	climatic niche distance mean	0.023	1	1.11	0.297
	$\Delta^{13}C$.RDPI	0.036	1	1.72	0.195
	$\Delta^{13}C$.mean	0.003	1	0.15	0.704
	ploidy:$\Delta^{13}C$.RDPI	0.007	1	0.36	0.553
	ploidy:$\Delta^{13}C$.mean	0.050	1	2.42	0.137
	R^2_m : 0.280				
	R^2_c : 0.563				

General linear mixed model specification:
model <- lmer((Fitness mean)$^{0.1}$ ~ Fixed effects + (1 | Nested random effects))
Fixed effects: climatic niche distance mean + trait plasticity + trait mean + ploidy:trait plasticity + ploidy:trait mean, where climatic niche distance mean and trait mean represent genotypic values averaged across all garden environments
Nested random effects: ploidy/taxon/population
The response variable (genotypic fitness averaged across all garden environments) of each LMM was power transformed (power parameter = 0.1). R^2_m, model marginal R^2 representing variance explained by fixed effects; R^2_c, model conditional R^2 representing variance explained by both fixed effects and random effects.

References

Bartoń K. 2017. MuMIn: Multi-model inference. R package version 1.40.40. https://CRAN.R-project.org/package=MuMIn.

Bates D, Machler M, Bolker BM, Walker SC. 2015. Fitting linear mixed-effects models using lme4. *Journal of Statistical Software* **67**: 1-48.

Ehleringer JR, Björkman O. 1978. Pubescence and leaf spectral characteristics in a desert shrub, *Encelia farinosa*. *Oecologia* **36**: 151-162.

Farquhar GD, Richards RA. 1984. Isotopic composition of plant carbon correlates with water-use efficiency of wheat genotypes. *Australian Journal of Plant Physiology* **11**: 539-552.

Hadfield JD. 2010. MCMC methods for multi-response generalized linear mixed models: The MCMCglmm R package. *Journal of Statistical Software* **33**: 1-22.

Hetherington AM, Woodward FI. 2003. The role of stomata in sensing and driving environmental change. *Nature* **424**: 901-908.

Lenth RV. 2016. Least-squares means: the R package lsmeans. *Journal of Statistical Software* **69**: 1-33.

Poorter H, Niinemets U, Poorter L, Wright IJ, Villar R. 2009. Causes and consequences of variation in leaf mass per area (LMA): a meta-analysis. *New Phytologist* **182**: 565-588.

Revelle W. 2017. psych: Procedures for personality and psychological research, Northwestern University, Evanston, Illinois, USA. R package version 1.7.8. https://CRAN.R-project.org/package=psych.

Sack L, Scoffoni C. 2013. Leaf venation: structure, function, development, evolution, ecology and applications in the past, present and future. *New Phytologist* **198**: 983-1000.

Schneider CA, Rasband WS, Eliceiri KW. 2012. NIH Image to ImageJ: 25 years of image analysis. *Nature Methods* **9**: 671-675.

Sletvold N, Ågren J. 2012. Variation in tolerance to drought among Scandinavian populations of *Arabidopsis lyrata*. *Evolutionary Ecology* **26**: 559-577.

Stamatakis A. 2014. RAxML version 8: a tool for phylogenetic analysis and post-analysis of large phylogenies. *Bioinformatics* **30**: 1312-1313.

Wright IJ, Reich PB, Westoby M, Ackerly DD, Baruch Z, Bongers F, Cavender-Bares J, Chapin T, Cornelissen JHC, Diemer M *et al.* 2004. The worldwide leaf economics spectrum. *Nature* **428**: 821-827.
